# Determination of spinal tracer dispersion after intrathecal injection in a deformable CNS model

**DOI:** 10.3389/fphys.2023.1244016

**Published:** 2023-09-25

**Authors:** Ayankola O. Ayansiji, Daniel S. Gehrke, Bastien Baralle, Ariel Nozain, Meenesh R. Singh, Andreas A. Linninger

**Affiliations:** ^1^ Department of Bioengineering, University of Illinois Chicago, Chicago, IL, United States; ^2^ Department of Chemical Engineering, University of Illinois Chicago, Chicago, IL, United States; ^3^ UIC Student Intern From EPF, Ecole D’Ingénieur, Paris, France; ^4^ Department of Neurosurgery, University of Illinois Chicago, Chicago, IL, United States

**Keywords:** geometry-induced mixing, intrathecal drug delivery, method of moments, oscillatory CSF flow, *in vitro* deformable spine model, drug infusion parameters

## Abstract

**Background:** Traditionally, there is a widely held belief that drug dispersion after intrathecal (IT) delivery is confined locally near the injection site. We posit that high-volume infusions can overcome this perceived limitation of IT administration.

**Methods:** To test our hypothesis, subject-specific deformable phantom models of the human central nervous system were manufactured so that tracer infusion could be realistically replicated *in vitro* over the entire physiological range of pulsating cerebrospinal fluid (CSF) amplitudes and frequencies. The distribution of IT injected tracers was studied systematically with high-speed optical methods to determine its dependence on injection parameters (infusion volume, flow rate, and catheter configurations) and natural CSF oscillations in a deformable model of the central nervous system (CNS).

**Results:** Optical imaging analysis of high-volume infusion experiments showed that tracers spread quickly throughout the spinal subarachnoid space, reaching the cervical region in less than 10 min. The experimentally observed biodispersion is much slower than suggested by the Taylor–Aris dispersion theory. Our experiments indicate that micro-mixing patterns induced by oscillatory CSF flow around microanatomical features such as nerve roots significantly accelerate solute transport. Strong micro-mixing effects due to anatomical features in the spinal subarachnoid space were found to be active in intrathecal drug administration but were not considered in prior dispersion theories. Their omission explains why prior models developed in the engineering community are poor predictors for IT delivery.

**Conclusion:** Our experiments support the feasibility of targeting large sections of the neuroaxis or brain utilizing high-volume IT injection protocols. The experimental tracer dispersion profiles acquired with an anatomically accurate, deformable, and closed *in vitro* human CNS analog informed a new predictive model of tracer dispersion as a function of physiological CSF pulsations and adjustable infusion parameters. The ability to predict spatiotemporal dispersion patterns is an essential prerequisite for exploring new indications of IT drug delivery that targets specific regions in the CNS or the brain.

## Background

Previous studies on intrathecal (IT) administration in pigs using very slow infusion rates (Bernards, 2006) contributed to the widely held belief that IT administration is confined to a small location near the injection site and, thus, is unsuitable for drug targeting of the brain. A further belief concerns the conjecture that IT drug delivery follows the phenomenon of solute dispersion in oscillatory pipe flow, known as Taylor dispersion in the engineering community (Tsangaris and Athanassiadis, 1985; Nelissen, 2008). However, the applicability of Taylor dispersion on drug transport in oscillatory cerebrospinal fluid (CSF) flow has not been tested *in vivo* due to technical difficulties and risk to patients. Tracking tracer dispersion *in vivo* with multimodal PET/MRI (Tangen et al., 2020) or computed tomography angiography suffers from limitations in spatial and temporal feature resolution (Figueiredo et al., 2012). In particular, observation of fast injection jets would require real-time acquisition rates unavailable in the current non-invasive imaging technology (Gholampour and Bahmani, 2021). Hence, technical limitations for tracking solutes suspended in complex CSF flow and patient safety render *in vivo* quantification problematic, if not impractical. *In vitro* experiments, using an anatomically accurate deformable model of the spinal subarachnoid space (SAS), are a complement and a logical alternative to authentic, but often inaccurate, *in vivo* infusion trials with limited temporal and spatial resolution. Several labs have also employed *in vitro* tests (Hettiarachchi et al., 2011; Bottan et al., 2012; Tangen et al., 2016a; Tangen et al., 2017a; Gholampour and Bahmani, 2021) and CFD models (Sweetman et al., 2011; Yiallourou et al., 2012; Tangen et al., 2015; Khani et al., 2018) for studying central nervous system (CNS) dynamics. IT *bench-top testing* with optical image analysis offers the distinct benefit of achieving high temporal and spatial resolution, which is necessary for systematic parameter studies of the correlation between infusion and physiological parameters (=anatomy and CSF dynamics) and achievable drug distributions. A key requirement for realistic infusion bench tests is the availability of an anatomically accurate model of the spinal microanatomy with a deformable, closed, fluid-filled spinal compartment with controllable pulsatile CSF flow.

In this paper, we will present parametric studies of IT infusion experiments in a subject-specific, anatomically accurate, deformable, and transparent replica of the human spinal SASs with natural CSF pulsations within the physiological range of spinal fluid amplitude and frequency inside the closed spinal compartment reproduced in a unique 3D printed, deformable, and transparent CNS analog. High-speed video recording enabled accurate observation of spatiotemporal tracer distribution patterns following high-volume IT injection as a function of natural CSF oscillations. The results characterize the speed of the tracer front (hereafter referred to as *dispersion speed*) as a function of infusion settings (infusion volume, flow rate, position, duration, and catheter diameters) and natural physiological properties (i.e., CSF stroke volume amplitude and frequency). We further compare the experimental data with prior theories (Taylor) of solute transport in oscillatory pipe flow.

## Materials and methods

### *In vitro* human spine and central nervous system model

**FIGURE 1:**
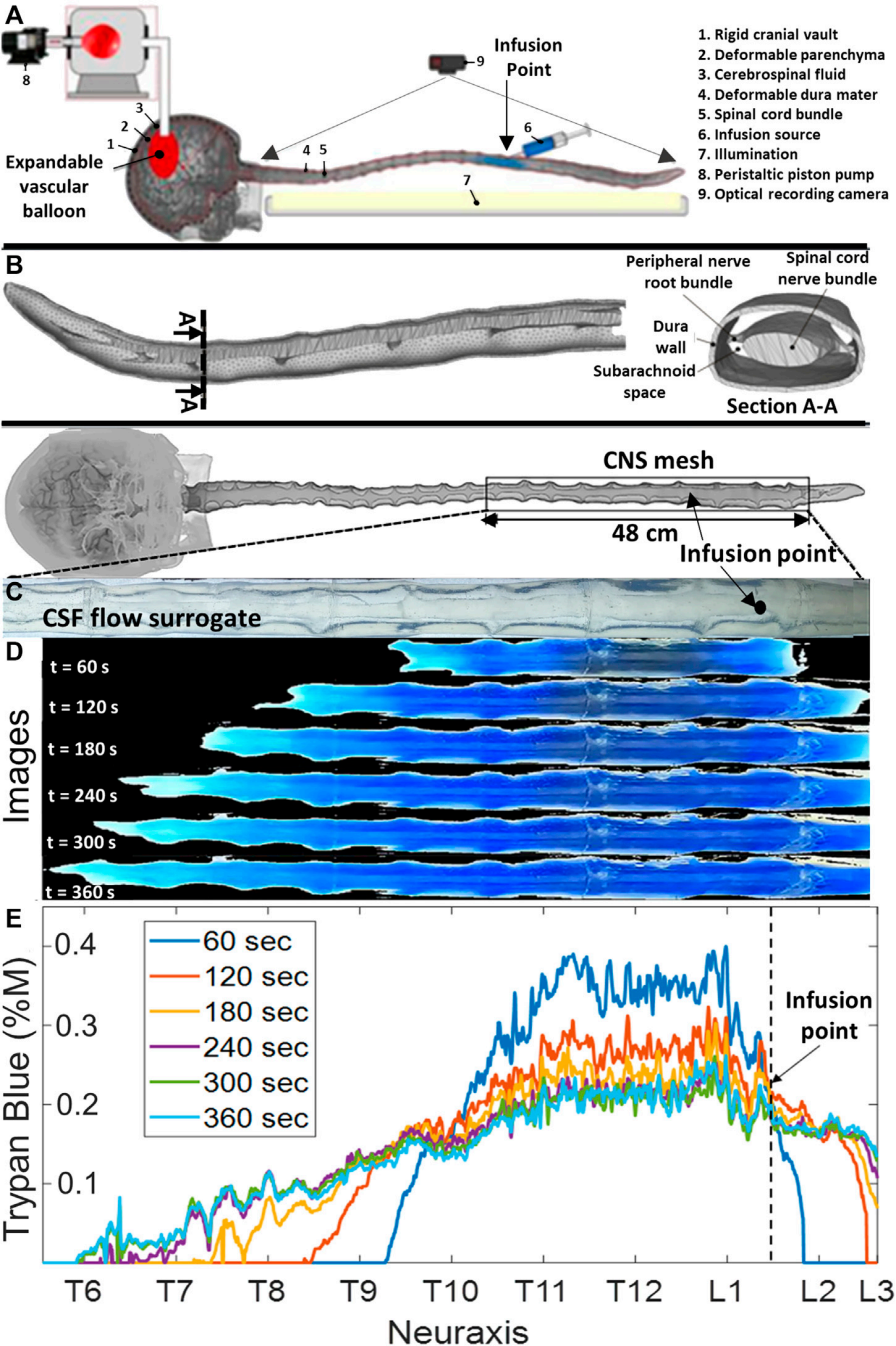
Phantom geometry and videography of the dispersion of the infused tracer in the central nervous system flow surrogate model. **(A)** Schematic diagram of the experimental setting. **(B)** Meshed model of a subject-specific central nervous system spine phantom model with peripheral nerve roots obtained from MR images. **(C)** Depiction of the optically clear cerebrospinal flow surrogate model before the injection (no blue dye is visible yet). **(D)** Progressing tracer front spreading from the injection site, preferably in the cranial direction. CSF: cerebrospinal fluid, IVF: infusion volumetric flowrate, and CNS: the central nervous system. **(E)** Overview of experiments and the time-lapsed images from a typical infusion experiment with IVF 2.0 mL/min, pulsation 1.0 mL/beat, and 0.4 %M of trypan blue concentration. It also shows a ruler with labels for the anatomical regions (i.e., cervical, thoracic, and lumbar) and the axial coordinate system along the neuroaxis.

We designed a deformable model of the human CNS to reproduce functional biomechanical relations between dynamically interacting CSF compartments (Figures 1A, B). An anatomically accurate analog of the spinal SASs with the transparent spinal cord including pairs of peripheral nerve roots and the translucent dural surfaces was manufactured in a multistep 3D printing and casting process with subject-specific imaging data of a 26-year-old male volunteer (Tangen et al., 2015). A mold was 3D printed using dissolvable polyvinyl alcohol (PVA). Platinum silicone casting resin of shore A8 hardness (TAP Plastics Inc.) was used for casting a flexible and transparent phantom. All parts including the dural and pial surfaces and nerve roots are deformable. Spinal CSF motion was generated by transmitting oscillatory expansion and contraction of an inflatable balloon located in the head section to the fluid. The balloon, mimicking the cerebrocranial vascular bed, in turn, was driven by a piston pump capable of generating stroke volumes up to 1 mL/beat in the frequency range of 0–127 beats per minute, as shown in detail in Supplementary Appendix SA. The connected spinal and cranial CSF spaces had no artificial openings consistent with the closed nature of the natural CNS. The mode of pulsatile flow conditions in the spinal CSF-filled spaces of the bench model reproduces pulsatile vascular bed dilation consistent with our understanding of periodic intracranial CSF displacement (Basati et al., 2012; Tangen et al., 2015). More manufacturing details of the subject-specific CNS replica (including 3D printing settings, stitching, and patching) can be found elsewhere (Tangen et al., 2016b; Lu et al., 2016; Ayansiji, 2023).

To reproduce conditions of *in vivo* IT procedures on the bench, infusion catheters with inner diameters of 0.2 mm, 1.0 mm, and 3.2 mm were inserted into lumbar and thoracic regions with inner diameters of 0.2 mm, 1.0 mm, and 3.2 mm. The elastic dura of the spine model has a self-sealing property, thus enabling realistic catheter insertion and placement as used in clinical practice for human therapies. A wide range of infusion parameter settings (i.e., infusion volume, flow rate, position, and duration) and systemic blood pump settings (i.e., stoke volume and frequency) enabled the implementation of a comprehensive spectrum of infusion scenarios (bolus and chronic drug pump) occurring in the physiological range of CSF oscillations. More than 50 tracer infusion experiments of trypan blue (Sigma-Aldrich) released with a programmable syringe pump (Harvard Instruments) were performed to precisely investigate the correlation between dye dispersion, CSF pulsations, and infusion parameters. Trypan blue was chosen for its intense blue color needed in optical front tracking. In a clinical setting, acute high-volume injection via a catheter is administered in a lying down (horizontal) position for safety. Thus, the horizontal patient position was chosen for our experiments.

### Tracer dispersion tracking with videography

#### Automatic image processing

Snapshots were obtained from the experimental videos showing the dispersion of the tracer at different times. Supplementary Appendix SB shows more details. MATLAB 2019b was used for semi-automated image analysis and quantification of dye dispersion. A filing system stored key parameters for each experimental run: infusion volume, infusion molarity, infusion volumetric flow rate (IVF), experiment duration, subject orientation (supine), oscillation frequency, and oscillation amplitude. Each video frame captured red–green–blue (RGB) data in the range of 0–255 for each pixel at location x and time. The RGB values were converted to grayscale and concentration to track the expansion of the dispersion front of the trypan blue, as shown in details in Supplementary Appendix SC. Analysis was divided into two phases: Phase-1 (acute infusion, t = 0–1 min) covered the time the infusion pump actively discharged dye into the CNS replica. Phase-2 (after infusion, t > 1 min) further tracked dynamic tracer spread under the influence of natural CSF pulsations (without further infusion). The results displayed in this work are of those obtained using the grayscale method and not the binary method.

#### Optical analysis of tracer concentration

RGB triplet values for pixels along the neuraxis were recorded in each frame used for analysis (typically snapshots 1 minute apart). The RGB color values were aggregated with a grayscale formula and intensities inferred from white-offset (white = no dye, blue = all dye). White-offset and area under the curve (AUC) scaling enabled quantitative dye concentration $C\left(x,t\right)$ inference at a particular position, x, along the neuraxis (Supplementary Appendix SC).

#### Analysis of the dispersion front

A custom image analysis code was used to determine the speed and spatial position of the visible dye front. Figures 1C, D show a time-lapsed series of images showing the expansion of the blue tracer before and after infusion at time points 0 s, 60 s, 120 s 180 s, 240 s, 300 s, and 360 s. Figure 1E shows tracer concentrations profiles inferred from the extracted RGB pixel intensity as described in *Materials and methods*. There was no tracer absorption into the dura and parenchymal walls that would have affected lighting properties. Trypan blue did not stain our system, and no loss of material was observed after months of use.

### Experimental determination of the speed of the advancement of the tracer front

The *method of moments* (MoM) was used to determine the apparent tracer dispersion velocity from video data because it minimizes the sensitivity of optically acquired concentration profiles to uneven lighting conditions, scattering effects, and uncertainty in concentration inference from intensity data (Birk et al., 2005). The MoM has been previously used to quantify dispersion in an annular tube (Mondal and Mazumder, 2005; Nagarani and sebastian, 2013; Debnath et al., 2019). Our experiment was insensitive to variations in lighting conditions, uncertainty of intensity quantification, and cardiac cycle synchronization; thus, we did not need gated acquisition. This is due to the advantage that MoM only tracks the slope of the evolution of moments over relative prolonged periods of time (=mean and variance of the entire intensity profile), so it is insensitive to perturbations of absolute concentrations or experimental variability. We first determined the *speed of caudocranial motion* of the infusate by tracking shifts in the center of gravity of the tracer profile. The first moment $m_{1}\left(t\right)$ of the observed concentration profiles at time t is calculated as follows:

$$m_{1}\left(t\right)=\frac{\int_{x_{o}}^{x_{m}}C\left(x,t\right)\;x\;dx}{\int_{x_{o}}^{x_{m}}C\left(x,t\right)\;dx}=\bar{x}\left(t\right),$$ (1)

where x is the position along the neuroaxis and $x_{0}$ marks the infusion point. The extreme limit of the ROI equal to full length from caudal to cranial aspects measured $x_{m}$ = 48 cm. In each time frame, t, the first moment, $m_{1}\left(t\right)$ , gives the location of the center of gravity, $\bar{x}\left(t\right)$ , of the tracer concentration profile at a time point, $C\left(x,t\right)$ . The *speed of caudocranial motion* is equal to the change of the first moment with time.

We further computed the second moment or area moment of inertia for each time point as as follows:

$$m_{2}\left(t\right)=\frac{\int_{x_{o}}^{x_{m}}{\left[x\left(t\right)-\bar{x}\left(t\right)\right]}^{2}\;C\left(x,t\right)\;dx}{\int_{x_{o}}^{x_{m}}C\left(x,t\right)\;dx}={\sigma \left(t\right)}^{2}.$$ (2)

The second moment can be interpreted as the mean spread of the visible concentration profile around its center, $\bar{x}\left(t\right)$ . The increase in its variance with time, ${\sigma \left(t\right)}^{2}$ , is directly proportional to the apparent dispersion or diffusion rate, $D_{exp}$ , of the tracer molecule as follows:

$$D_{exp}=\frac{1}{2}\;\frac{∆{\sigma \left(t\right)}^{2}}{∆t}.$$ (3)

The coefficient of the apparent dispersion can then be readily determined as the rate of change in the second moment of the concentration curves by plotting their variance as a function of time. Supplementary Appendix SD shows details.

### Stroke volumes and mean pulsatile flow velocities for formal analysis

In MR imaging, it is convenient to characterize natural CSF pulsations via the instantaneous total CSF volume, V(t), cervical stroke volume, $v_{c}$ , and CSF angular pulse frequency, ω . We estimated the average CSF flow velocity, U_rms_, as follows:

$$U_{rms}=\sqrt{\frac{\int_{0}^{t}{\left(V\left(t\right)/A\right)}^{2}}{T}dt}\;V\left(t\right)=V_{0}+\left(\frac{v_{c}}{2}\right)-\left(\frac{v_{c}}{2}\right)\cos\left(\omega \;t\right),A=\frac{V}{L},\omega =2\pi \;N_{bpm}.$$ (4)

The average CSF flow velocity, U_rms_, correlates a clinical MR quantity to parameters used in our formal flow analysis. Thus, two stroke volume settings of 0.5 mL/beat to 1 mL/beat in the frequency range of 0–120 beat/min enabled us to cover a wide range of CSF flow velocity averages covering the entire physiological range (0.09–0.93 cm/s).

The mean CSF flow velocity, $U_{rms}$ , in the cervical region was computed by integrating and averaging the squared ratio of pulsatile volumetric flow rate, $\frac{dV\left(t\right)}{dt}$ , divided by the hydraulic cross-sectional area of the spinal CSF SAS, A, under cosine profile assumption as in Eq. 4, where V_0_ is the initial volume of CSF in cm^3^ in the system and f is the frequency in beats per minute, with period T in seconds.

$$U_{rms}=\sqrt{\frac{\int_{0}^{t}{\left(V\left(t\right)/A\right)}^{2}}{T}dt}\;V\left(t\right)=V_{0}+\left(\frac{v_{c}}{2}\right)-\left(\frac{v_{c}}{2}\right)\cos\left(\omega \;t\right),A=\frac{V}{L},\omega =2\pi f.$$

### Statistical analysis

Initially, the experiments were repeated three times with the same settings. The repeats essentially gave the same moments, thus confirming the reproducibility and robustness of data acquisition. The results of the Shapiro–Wilk test indicated that all datasets had a normal distribution. The ANOVA test was used in the regression analyses using IBM SPSS v26 to confirm that regression models described the dependent variables. Confidence intervals were calculated for all regression results. The Durbin–Watson statistic for all regression results was 0.716; therefore, the datasets have autocorrelation. Furthermore, calculated tolerances indicated the multicollinearity effect in the datasets. A *p*-value of 0.05 was considered statistically significant.

## Results

### Dynamic tracer profile front tracking

**FIGURE 2:**
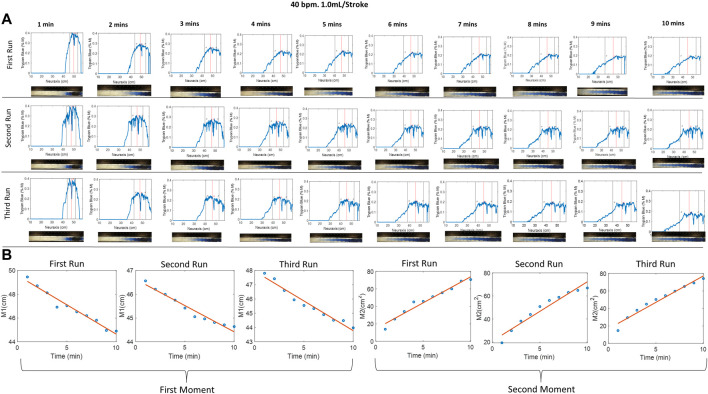
**(A)** Evolution of the tracer at different times of 40 bpm, 1.0 mL/stroke at different runs, where the red and black vertical lines represent the mean and variance of the curve, respectively. Img means image. **(B)** The first and second moment for the three identical experiments. The small difference between repetitions suggests acceptable experimental variability. The first moment, M1, indicates that the tracer moves in the cranial direction from the injection position around (50 cm) toward the thoracic region position around (44 cm). The second moment characterized the apparent speed of tracer dispersion. Half of the slope of the second moment plot gives the dispersion coefficient.

Figure 2A shows tracer concentration profiles inferred from calibrated RGB pixel intensity as described in Materials and methods. Figure 2B shows the first and second moment for the selected experiment. The first and second moments for all the experiments are given in Supplementary Appendices SC, SD. The close agreement between three repetitions (=variance of the second moment ±0 .29 cm^2^/min) confirms the reproducibility and acceptably low experimental variability of the experimental setting. The first moment gives information about the caudocranial velocity, and the second moment determines the dispersion coefficient of the tracer as shown in Eq. 3. The binary mask was used to crop images to the width of the spinal section covered by detectable tracer intensity. The size of this mask along the neuraxis (x-direction) is referred to as the *dispersion width* of the tracer at time t, DD(t). The next section shows the systematic analysis of infusion and physiological parameters on the speed of tracer dispersion.

### Effect of catheter diameter on initial tracer spread during acute infusion (phase-1)

We studied the effect of catheter diameter on the speed and size of the dispersion front. Infusion experiments are performed using three different catheters with inner diameters of 0.2 mm (needle N1), 1.0 mm (N2), and 3.2 mm (the widest needle N3). Infusion lasted 1 min over the course of 54 experiments. The dispersion width after 10 min, DD_10_, was measured from the point of the needle tip to the tip of the dye front in the caudal and cranial directions. The duration of 10 min was chosen because this initial time window is critically important for assessing acute risks associated with high-volume IT injection. High local toxicity has been implicated with granuloma formation (Kaye et al., 2004; Allen et al., 2006). We also varied infusion volumetric flow rates (IVF = 0.5, 1.0, and 2.0 mL/min).

**FIGURE 3:**
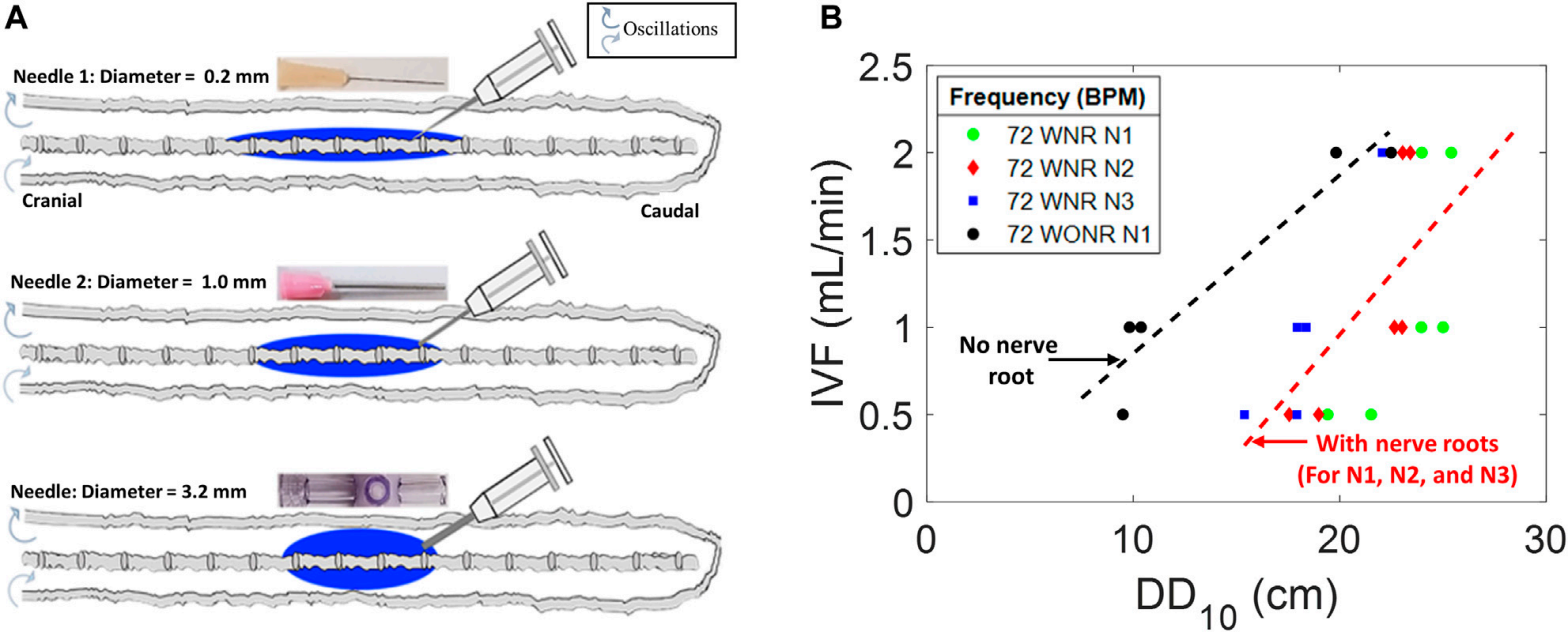
Effects of needle diameter, IVF, and DD on tracer dispersion. **(A)** Central nervous system spine model is shown as a diagram with varied diameter infusion catheters and corresponding idealized dispersion patterns (blue). **(B)** Relation of the infusion type to DD_10_ and needle diameter in a CNS spine model with peripheral nerve roots (colorful) and without peripheral nerve roots (gray). DD_10_ represents the linear dispersion distance in the tracer front after 10 min of infusion. N1, N2, and N3 represent needles 1, 2, and 3, respectively. IVF: infusion volumetric flow rate. WNR: with nerve root. WONR: without nerve root.

Figure 3A shows that high-caliber catheters promote slower tracer spread during infusate injection (1 min). We observed the formation of an injection jet, which was less pronounced in high-caliber catheters leading to shorter dispersion width after 1 min, DD_1_. For identical infusion flow rate (IVFs from 0.5, 1.0, and 2.0 mL/min), the large caliber needle (N3) generated the shortest dispersion width. Figure 3B shows the effect of inner catheter diameter on the extent of the tracer spread observed 10 min after the infusion as a function of infusion flow. All experimental results (N = 54) were also fitted into a linear regression model that can be used to estimate the initial neuraxial coverage (=size of the region dosed by solute) as a function of catheter lumen and infusion volumes (IVF). The regression model

$$DD\;\left(10\;\min\right)=a\left(ND\right)+b\left(IVF\right)+c$$ (5)

can serve as a guideline in the clinical practice to estimate the length of initial dispersion as a function of catheter lumen and IVF (Figure 3B), where ND is the inner needle diameter and a,b,and c are −1.4150, 2.8786, and 19.8668, respectively, with an R^2^ value of 0.7916.

The realization of a fixed infusion rate with thinner catheters requires higher infusion pressure resulting in higher exit velocities ( $V_{Ext}$ ) at their tips, leading to the creation of an injection jet in the close vicinity of the catheter tip. Thus, catheter N1 at IVF = 2.0 mL/min has the largest $V_{Ext}$ and kinetic energy ( KE ) (as shown in Supplementary Appendix SA). Also, the exit velocity and kinetic energy of catheter N1 are higher than those of needles 2 and 3 with the same IVF. In thin catheters, a higher infusion impulse is delivered during the injection phase that translates into a wider dispersion length in the observed tracer front for the same injection flow rate.

### Effect of injection parameters on initial caudocranial dispersion (phase-1, acute infusion)

**FIGURE 4:**
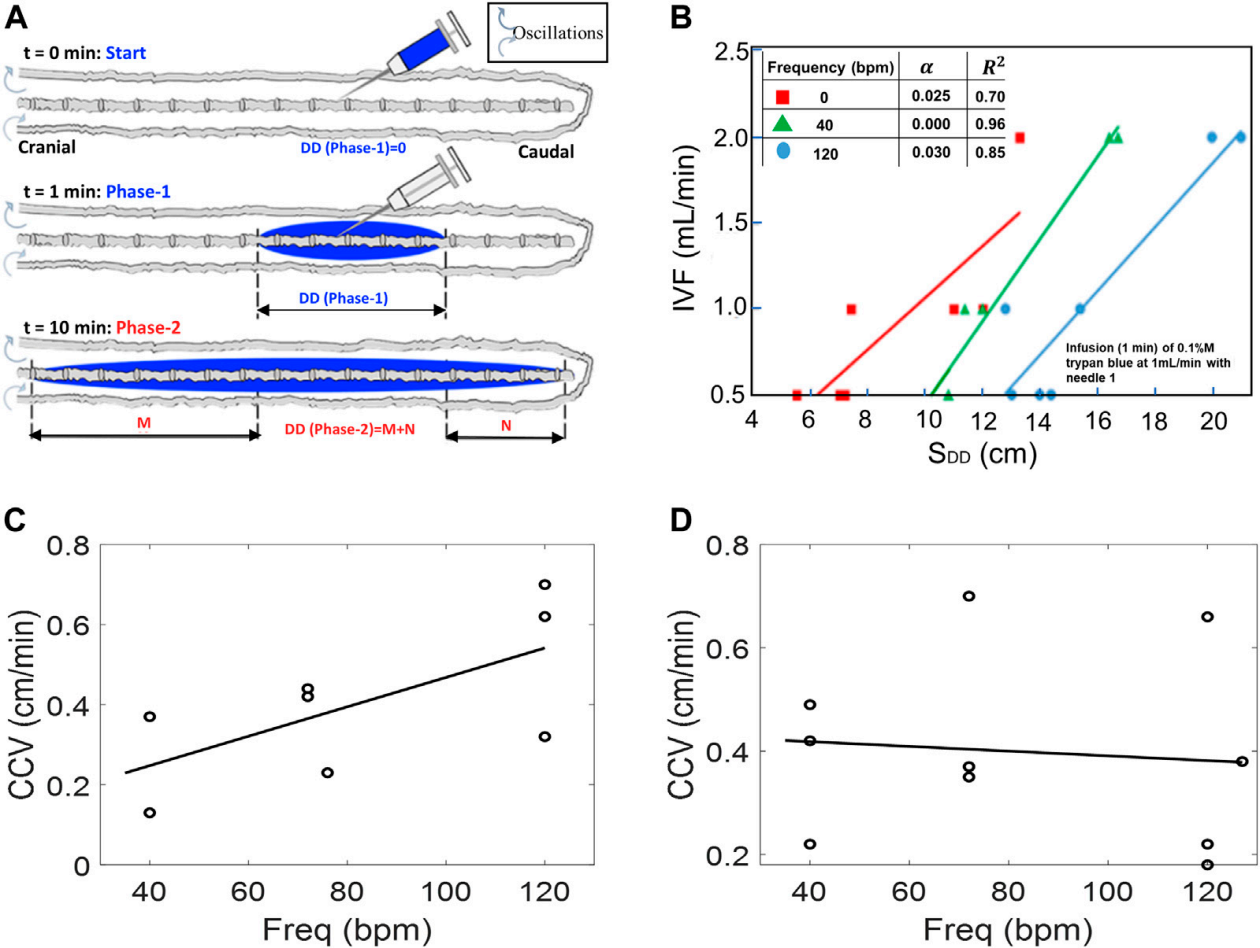
**(A)** Schematic depicting experimental setting in phase-1 tracer dispersion experiments. All results pertain to injection phase (phase-1). **(B)** Effect of CSF oscillation frequency on dispersion length S_DD_ (with nerves) using IVF of 0.5, 1.0, and 2.0 mL/min under oscillation frequencies 0, 40, and 140 bpm, respectively. **(C,D)** Caudocranial velocity (CCV) changes of the tracer relative to frequency for pulsation for 0.5 mL/beat **(C)** and 1.0 mL/beat **(D)**. During the infusion phase, CCV is not frequency dependent, especially at higher CSF amplitudes.

Experimental settings of the injection flow rate, injection volume, and catheter specifications were varied to explore optimal conditions for targeting the cervical section or brain area. Fifty-four experiments (N = 54) were conducted to systematically characterize tracer targeting toward the cranial compartment as a function of infusion parameters (phase-1, shown in Figure 4A). The results in Figure 4B show that higher infusion flow rates IVF accelerate the speed of the tracer front advancing in the cranial direction from the infusion catheter tip. The distance the tracer front moves in the cranial direction from the infusion catheter tip for the CNS model in the supine position as observed 10 min after the infusion is represented by S_DD._ This effect at high-volume injections is due to the increased insertion kinetic energy (Supplementary Appendix SA).

The *speed of caudocranial motion*, CCV, is calculated as the change of the first moment with respect to time as shown in Figure 2B. All experiments showed a shift of the first moment toward the cranium. We determined the velocity of apparent caudocranial advancement of the tracer front, CCV , by recoding its positions for different time points. The results of Figure 4C show that the caudocranial advancement of the tracer front from the injection site toward the cranium slightly increased with oscillation frequency, f , as indicated by the regression

$${CCV}_{f}\;\left(cm/\min\right)=a\;f+b$$ (6)

and $U_{rms}$ **,** as root-mean-square velocity, as indicated by the regression

$${CCV}_{U_{rms}}\;\left(cm/\min\right)=a\;U_{rms}+b$$ (7)

with constant terms a and b as 0.3014 and 0.0012 for Eq. 6 and 0.3752 and 0.0012 for Eq. 7, respectively. At 1/mL stroke volume, this trend was similar but with a wider variability between runs (Figure 4D).

The rapid expansion of the drug front follows the amount of infused tracer qualitatively, as expected, because the main driver of the initial tracer spread is the injection impulse of fresh infusate concentrated in the relative narrow spatial confinement in the lumbar injection zone.

### Dispersion by natural CSF pulsation (phase-2, after injection)

**FIGURE 5:**
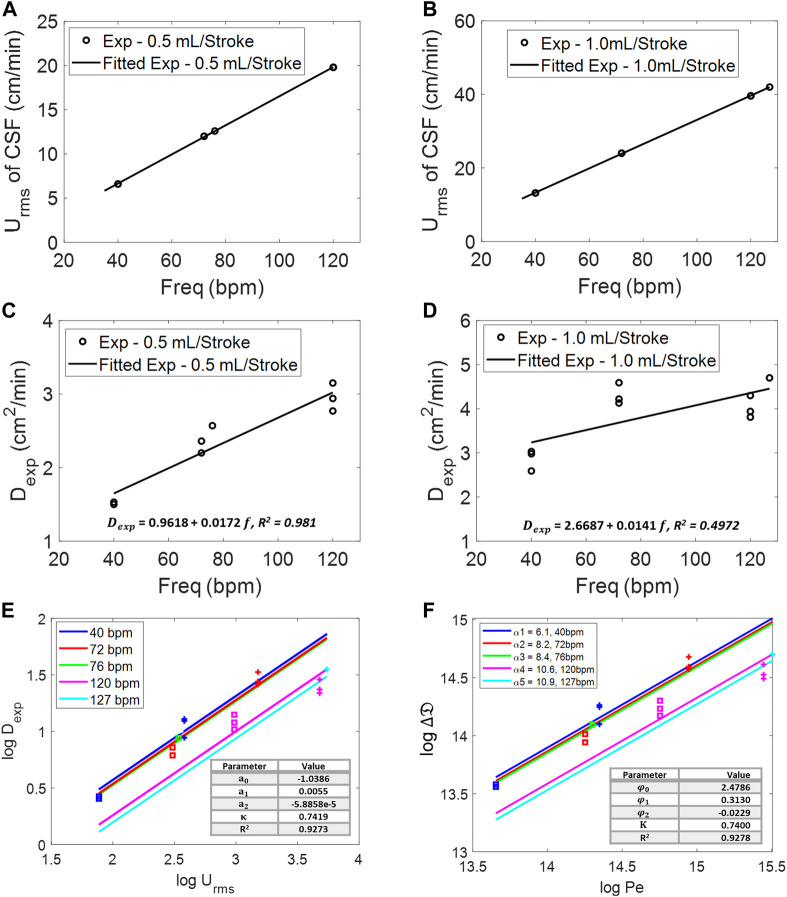
Root-mean-square velocity of the CSF at different frequencies for 0.5 mL/beat **(A)** and 1.0 mL/beat **(B)**. Experimental diffusivity of infusion at different frequencies for **(C)** 0.5 mL/beat and **(D)** 1.0 mL/beat. **(E)** Log–log relation of root-mean-square velocity, U_rms_, of the CSF and experimental diffusivity, $D_{\exp}$ . Frequency dependence is added parametrically as straight lines for 40, 72, 76, 120, and 127 bpm. **(F)** Dimensionless log–log relation for the change in experimental diffusivity, ∆D , in terms of the Peclet number (amplitude) and Womersley number, α (frequency), of the CSF oscillatory flow. $D_{0}$ is the molecular diffusivity of the tracer (trypan blue $D_{0}=1.938\;X\;10^{-6}\;{cm}^{2}/\min$ ).

Once infusion stops (t > 1 min), further tracer spread is no longer propelled by injection impulse, but by natural oscillations of the CSF. In our closed, deformable model, induced CSF flow in the spinal SAS is also oscillatory with zero net flux. This is also approximately the situation *in vivo* since net flows due to bulk CSF production are much smaller than those due to oscillations. The apparent dispersion coefficient of tracer spread in the oscillatory CSF flow was determined experimentally as a function of amplitudes (cervical CSF stroke volume and mean CSF flow velocities) and frequencies in a series of dynamic tracer infusion experiments. Two stroke volume settings of 0.5–1 mL/beat in the frequency range of 0–120 beat/min enabled us to induce a wide range of CSF flow velocity averages covering a wide physiological range (U_rms_ = 0.09–0.93 cm/s). The root-mean-square velocity of the CSF, $U_{rms}$ , in the cervical region was computed as described in Materials and methods with plots as the function of frequency for a stroke volume of 0.5 mL/stroke and 1.0 mL/stroke in Figures 5A, B. Tracer dispersion reached the cervical region in less than 10 min, and it spread quickly throughout the spinal SAS. Figures 5C, D show dispersion coefficients $\;D_{\exp}$ determined by MoM (see Materials and methods) as a function of frequency for stroke volume of 0.5 mL/stroke and 1.0 mL/stroke experiments, respectively. Experimentally obtained (=MoM) dispersion coefficients, D_exp_, summarized in Figure 5 confirm our previous finding (Hsu et al., 2012; Hsu and Linninger, 2013) that CSF amplitude and frequency are critical factors of IT drug dispersion with the range of D_exp_ = 1.50–2.94 cm^2^/min for a 0.5 mL/beat stroke volume, and D_exp_ = 2.57–4.70 cm^2^/min for the 1 mL/beat stroke volume as shown in Table E2 and E3, respectively, of Supplementary Appendix SE. As the frequency increases, trypan blue disperses faster and ascends in cranial direction.

To derive functional correlations, Figure 5E lists experimental dispersion coefficient, D_exp_, as a function of natural CSF oscillations in terms of amplitude, $U_{rms}$ , and frequency, f , on a double logarithmic scale according to Eq. 8. The trend in double logarithmic scale, ${\log(D}_{\exp})∼\kappa$ *log* (U_rms_), had a constant slope of κ = 0.7419. A quadratic frequency dependence was also incorporated as an *f*-dependent offset, with $\lambda \left(f\right)=a_{0}+a_{1}\;f+a_{2}f^{2}$ with $a_{0=}-1.0386,a_{1}=0.0055,\text{and }a_{2}=-5.8858\;x\;10^{-5}$ , and drawn parametrically in Figure 5E. Taking advantage of the logarithmic form of Eq. 8, all parameters could be estimated simultaneously by linear regression with the coefficient of determination for the best fit of R^2^ = 0.9273.

$${\log(D}_{\exp})=\lambda \left(f\right)+\kappa \;\log\left(U_{rms}\right).$$ (8)

### Is IT distribution predicted by Taylor–Aris dispersion?

We also cast all experimental results into a dimensionless form for comparison to prior transport theories. We depart from the commonly known Taylor theory (Aris, 1960) and Aris (Salerno et al., 2020), which describes the increase of the effective diffusivity over molecular diffusivity, D_0_, as a function of the dimensionless Peclet number, $Pe=\frac{U_{r.m.s}*h}{D_{0}}$ , as in Eq. 9. Taylor dispersion in stationary flow has λ=1/48 and κ=2. Hoagland and Prud’Homme (1985) modified Taylor’s approach for laminar oscillatory flow within rigid cylindrical walls citing the coefficients, λ=0.0104 and κ=1.88.

$$\frac{D_{exp}}{D_{0}}=1+\lambda \;{Pe}^{\kappa }.$$ (9)

Figure 5E shows a slope in our experiments ( κ = 0.7419), which does not agree with the dispersion models by Taylor (1953) and Hoagland and Prud’Homme (1985). This discrepancy can be illustrated more clearly with the help of Eq. 10, which was obtained by rearranging Eq. 9. Accordingly, dimensionless effective dispersion increases, $∆D=\frac{D_{\exp}-D_{0}}{D_{0}}$ , were plotted in Figure 5F based on the raw dimensional data of Figure 5E. Again, the logarithmic correlations permit linear fitting for quantifying the dependence of effective dispersion increase, $\log\left(∆D\right)$ , on the dimensionless oscillatory flow amplitude (Peclet number) and dimensionless frequency (Womersley number, α ). Here, the Womersley number is $\alpha =\frac{D_{H}}{2}\sqrt{\frac{\omega }{\upsilon }}$ , $D_{H}=0.5cm$ is the hydraulic diameter, $\omega =\frac{2\pi f}{T}$ , f is the frequency, T=1 sec is the period of oscillation, and $\upsilon =7x10^{-7}$ m^2^/sec is the kinematic viscosity. All values are listed in Supplementary Appendix SE.

$$\log\left(∆D\right)=\lambda \left(\alpha \right)+K\;\log\left(Pe\right).$$ (10)

The best-fit parameter set ( Κ = 0.74, $\varphi_{0}=2.4786,\varphi_{1}=0.3130,\text{and }\varphi_{2}=-0.0229$ ) in Eq. 11 has a coefficient of determination of R^2^ = 0.9278.

$$\log\left(∆D\right)=\left(\varphi_{0}+\varphi_{1}\;\alpha +\varphi_{2}\alpha^{2}\right)+K\;\log\left(\text{Pe}\right).$$ (11)

**FIGURE 6:**
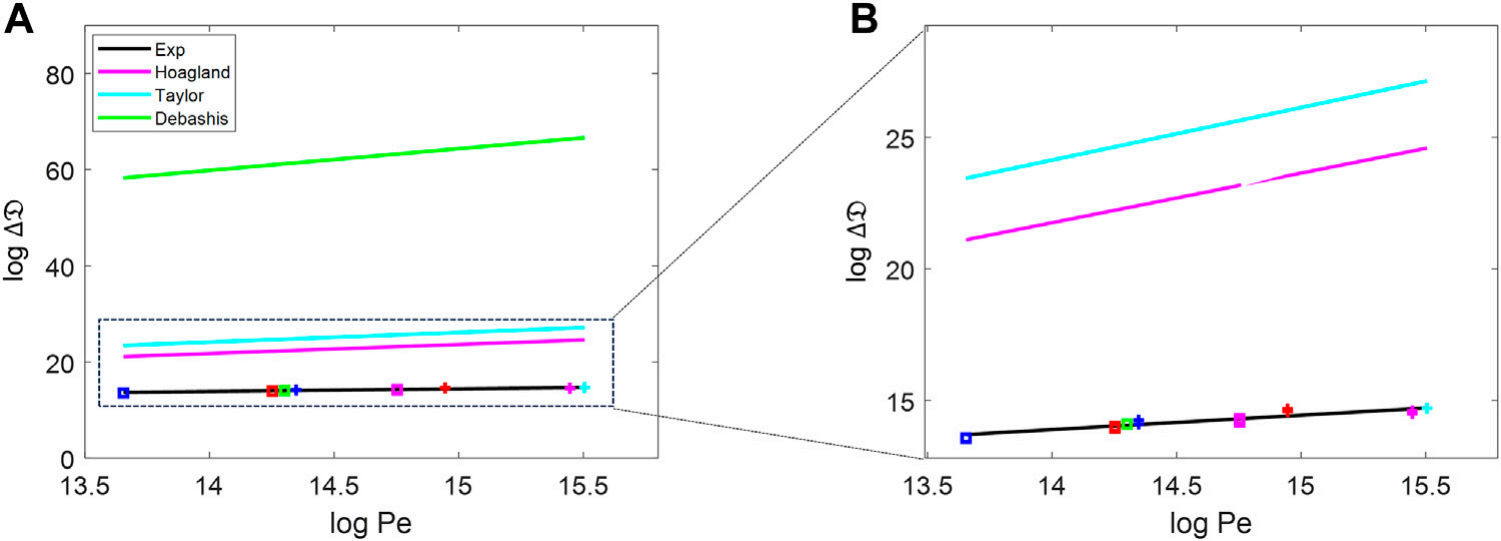
**(A)** Comparison of the dispersion change against the Peclet number between the experiment data, pure diffusion, and the prediction by Taylor approaches [Hoagland and Prud’Homme (1985), Taylor (1953), and Dutta and Leighton (2002)]. **(B)** Zoomed-in version of the experimental, Taylor, and Hoagland portions of **(A)**. The blue-, red-, green-, magenta-, and cyan-colored data points are for the frequencies of 40, 72, 76, 120, and 127 bpm, respectively. The data represented as square are for 0.5 mL/stroke, and those represented with + are for 1.0 mL/stroke.

We compared our experimental dispersion coefficient trends to prior work, including the work of Hoagland and Prud’Homme (1985) and Dutta and Leighton (2002). Figure 6A shows that several versions of Taylor–Aris dispersion (TAD) grossly overestimate the speed of biodispersion in the human spinal SAS. Figure 6B shows the zoomed view of the experimental portion of Figure 6A for better visibility. The Taylor (1953), Hoagland and Prud’Homme (1985), and Dutta and Leighton (2002) versions do not match our experiments even qualitatively because the slopes of the Pe dependence ( κ=1.88−2.0 ) are quite different ( κ = 0.7419, Κ = 0.74) in addition to the estimated value ranges being largely off.

We also compared our experimental values to theoretical dispersion coefficient estimates by Stockman (2007), who used Lattice Boltzmann simulations. Stockman used the maximum average speed in a period of oscillation of 220.2 cm/min (in lattice unit of 1 × 10^−2^ Lu/ts), and the molecular diffusion of the tracer used was D_0_ = 0.0024 cm^2^/min. The range of the determined dispersion coefficients was D = 0.6356–1.7735 cm^2^/min, which is much smaller than that found in our experiment by a factor of over 2.5. The Stockman data are also plotted in Supplementary Appendix SF.

In summary, Stockman’s (2007) results underestimate the speed of tracer dispersion, and the results of Hoagland and Prud’Homme (1985), Dutta and Leighton (2002), and Taylor (1953) overestimate the dispersion coefficient by three orders of magnitude or more compared to our *in vitro* data.

### Effect of nerve roots on dispersion distance in phase-2

We previously observed that annular phantoms without microanatomical features underestimate the actual dispersion after IT (Barton, 1983; Banyte et al., 2013). To test the significance of microanatomical features on tracer dispersion, we also fabricated a spinal model without nerve roots and compared tracer propagation during infusion to the more anatomically realistic model with nerve roots. Tracer dispersion in the model with nerve roots was found to be always much more rapid than in a system lacking nerve roots under the same condition of oscillatory flow (Figure 3B). Complex flow and observable mixing patterns are absent in idealized annular models lacking spinal microanatomy (Aris, 1960; Tsangaris and Athanassiadis, 1985; Salerno et al., 2020), thus failing to boost the effective dispersion of IT-injected tracers as this is the case when microanatomical features are present. The results of this study provide further evidence for the significant impact of microanatomical features on the spatial and temporal dispersion patterns of IT-administered solutes shown previously (Tangen et al., 2015; Haga et al., 2017).

### Reduced-order pharmacokinetic model for IT drug biodistribution

Experimental data for tracer infusion experiments served as the input for a reduced-order pharmacokinetic model of IT administration. The full description of this mechanistic drug administration model is beyond the scope of this manuscript but can be found elsewhere (Tangen et al., 2017a; Linninger et al., 2023). In brief, tracer biodispersion after lumbar intrathecal injection was simulated via a convection–diffusion process distributed along the neuraxis in Eq. 12. The effect of geometry-induced mixing due to natural CSF pulsations was captured via the effective diffusivity, $D_{eff}$ , determined in experiments described in Materials and methods and Results. Sensitivity of predictions to infusion settings was incorporated via a single source term, $V_{\text{inj}}\left(x,t\right)$ . The effect of injection flow rate, volume, and duration on the forces between infusate, spinal CSF interacting with deformable subarachnoid spaces (=dura) was accounted for by biomechanical fluid structure interaction (FSI). Accordingly, high-volume injections generate a non-zero caudocranial convection term, $U_{fsi}$ , during infusion (phase-1). After the infusion stops (phase-2), the tracer continues to spread away from the lumbar injection site in accordance with effective dispersion as shown in Eq. 12.

**FIGURE 7:**
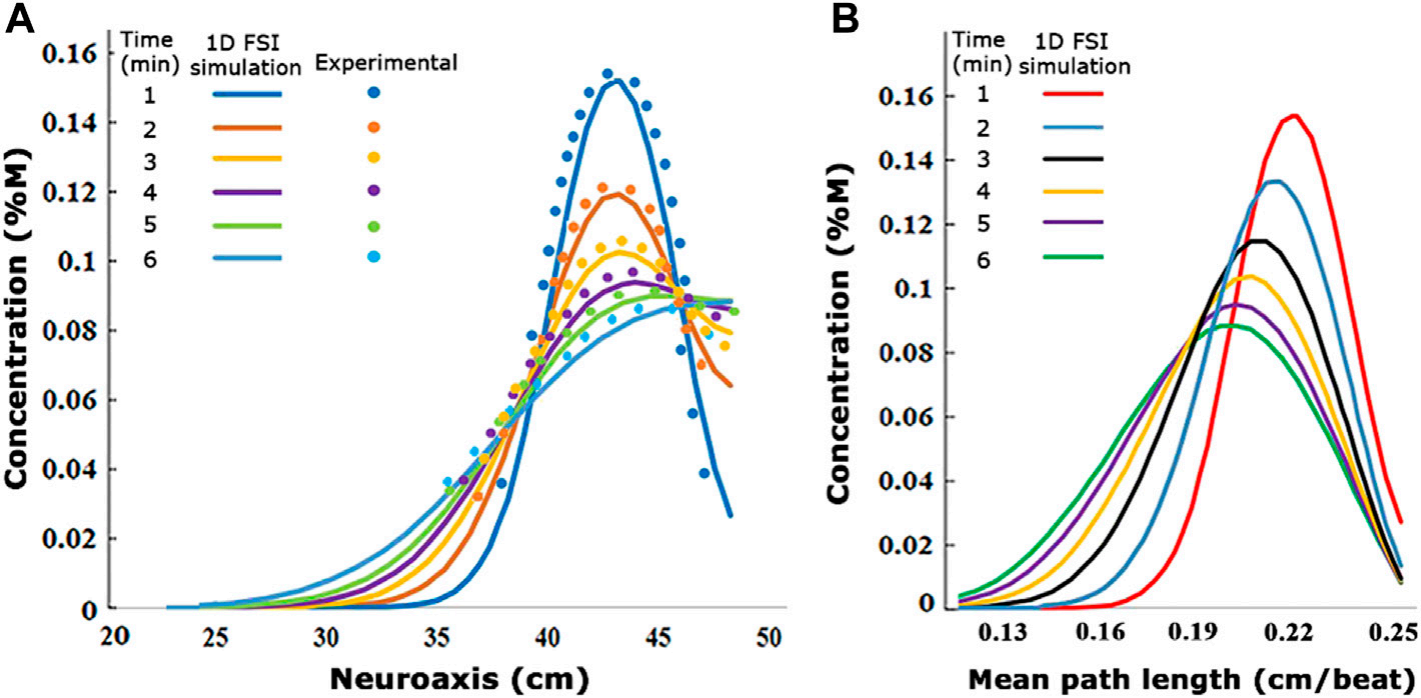
**(A)** Visualization of the experimentally derived concentration values compared to the 1D fluid–structure interaction simulation concentration values at different times. **(B)** Changes of 1D FSI simulation concentration diagrams in the different mean path lengths. The values are from infusion experiment parameters in 0.5 mL/beat .

Tracer concentration profiles along the neuraxis as a function of time were predicted with mechanistic pharmacokinetic simulations. Pharmacokinetic tracer profile simulations with CNS dimensions and infusion settings used in the experiments took less than one CPU minute to converge, generating asymmetrical profiles with peak concentration decreases with time (Figure 7A). The comparison with the experimental run shows a qualitative match both in the spatial and the temporal dimension as shown in Figure 7A. Figure 7B shows the maximum concentration is attained in a short interval of mean path length. The preliminary results of the proposed order model show that the use of experimentally obtained effective dispersion coefficients can effectively predict drug dispersion after IT administration using a reduced-order pharmacokinetic simulation at low computational cost. It is worth noting that the simulation of biodistribution of active drugs into the CNS and the systemic circulation required additional information on biochemical parameters, denoted by the sink term $R\left(c,x\right)$ , in the following equation:

$$\frac{\partial C\left(x,t\right)}{\partial t}=\vec{\nabla }\;\left[D_{eff}\vec{\nabla }C\left(x,t\right)\right]-U_{fsi}\left(x,t,V_{\text{inj}}\right)\;\vec{\nabla }C\left(x,t\right)+n_{\text{inj}}\left(x,t\right)-R\left(c,x\right),$$ (12)

where C is the concentration, $D_{eff}$ is the effective diffusion coefficient, t is the time, x is the position along the neuroaxis, $V_{\text{inj}}$ is the volume of injection, $U_{fsi}$ is the caudocranial convection, and $n_{\text{inj}}$ is the number of moles. The reaction rate expression might incorporate drug half-life, tissue uptake, and clearance (Tangen et al., 2015; Linninger et al., 2023).

## Discussion

### Realistic replica of the human CNS

IT infusion studies under physiological conditions require deformability of the CSF spaces to accommodate realistic pulse pressure propagation and fluid motion inside a closed spinal SAS. Deformable fluid-filled spaces in the present bench test analog of the human CNS is fully enclosed between the soft dura/parenchyma surfaces and a distensible vascular interface (cranium vault with a distensible vascular balloon) without fluid exchange across the open system boundaries. The proposed configuration approximates the anatomy and fluid–structure interaction dynamics of the spinal SAS, so we have confidence that it reproduces the complex geometry-induced CSF mixing patterns that were reported in the previous work using the direct numerical simulation of spinal CSF flow (Tangen et al., 2015). The CNS model also incorporates microanatomical features of the spinal cord, epidural space, and peripheral nerve root bundles, especially, which are critical geometric aspects implicated with enhanced mixing (Yiallourou et al., 2012; Pahlavian et al., 2014) of fluid layers in the spinal SAS, leading to accelerated drug dispersion.

Moreover, transparent borders of the see-through human CNS replica enabled dynamic optical tracking of tracer concentration profiles during and after IT solute administration. Our experiments characterized in detail two stages occurring during high-volume IT infusion. The initial phase during which the drug is injected lasts only a few minutes in clinical settings. The duration of 10 min was chosen because it is suitable for assessing acute risks associated with high-volume IT administration (local toxicity and granuloma formation).

### Injection phase

The axial dispersion during the injection phase (phase-1) correlated with infusion volume, IVF, and catheter diameters. Thinner catheters (inner diameter d = 0.2 mm) generate wider and fast initial solute dispersion as is attainable with high-caliber catheters (di = 3.2 mm inner diameter). Based on our series of experiments, a simple formula in Eq. 5 predicts the initial dispersion width, DD_10_, the distribution length after 10 min of injection, as a function of injection volume/flow rate and catheter diameter. The formula in Eq. 5 may serve to estimate the initial volume of distribution, peak concentrations, and initial neuronal tissue exposure during the acute infusion phase of high-volume drug administration as a function of catheter lumen and infusion flow rate. The peak local toxicity risk may be elevated in infusion protocols generating narrower initial spread (i.e., high-volume infusion with large caliber catheters). It can also be used to estimate the expected local volume of drug action and local drug concentration to assess the risk of granuloma formations (Allen et al., 2006).

### Drug pumps

Drug pumps are often deployed for the local treatment of chronic pain or spasticity but are less suitable for targeting the brain, which was one of the main objectives of the current study. Drug pumps operate under much lower flow rate settings than possible with acute administration. Accordingly, the effect of the injection impulse may be negligible. Chronic IT administration with drug pumps are, thus, predominately governed by conditions of natural oscillation (phase-2) for its entire time course as discussed as follows. For more recent developments for effective infusion protocols from drug pumps, we refer to the work by Yaksh et al. (2012) and Hildebrand et al. (2019).

### Dispersion in the naturally oscillating CSF (after the injection subsides)

After the injection ceases, the tracer spreads due to natural CSF pulsations. Oscillatory fluid flow around microanatomical features create a geometry-induced mixing pattern, which breaks the laminar flow field by introducing eddies and vortices around nerve roots. Localized and interspersed eddies were observed in the flow directly upstream and downstream of the cylindrical peripheral nerve root bundles suspended in the flow. Moreover, trabeculae can substantially enhance this effect as reported in Eq. 12.

### Caudocranial motion

In all infusion experiments, slow but steady caudocranial advancement of the tracer front from the lumbar injection site toward the cranium was observed*.* We offer two explanations for the experimentally observed caudocranial transport.

First, lumbar injection divides the space available for the solute to disperse into a smaller distal volume containing the sacral compartment and a larger cranially facing domain stretching from the catheter tip to the thoracic, the cervical, and the cranial SAS. We observed that tracers initially spread equally in both sections, which is consistent with a diffusive process. Subsequently, the advancing dye fronts fill the closed sacral domain faster due to its smaller size. Once the closed sacral region is occupied, the tracer profile center of gravity begins shifting toward the head. The asymmetric caudocranial tracer profile develops due to a boundary effect that was confirmed with a mechanistic diffusive transport model. Systematic experiments with varying CSF conditions (amplitude and frequency) enabled the determination of a formula for the velocity of the caudocranial shift as a function of CSF pulsations and frequency as shown in Eq. 6.

The graded biomechanical deformation profile of the spinal SAS responsible for CSF pulse attenuation is a secondary factor. The CSF pulse amplitude was shown to diminish gradually from the cervical toward the sacral region (Tangen et al., 2015). The CSF pulsations and average oscillatory CSF velocities, U_rms_, become larger toward the cervical region compared to the lumbar region where the pulse amplitude is almost zero. Thus, effective dispersion tends to become faster in the caudocranial direction. These two effects cannot be studied in rigid, open systems.

The infusion experiments were conducted in a closed deformable CNS model with no net CSF generation or removal. This supports the notion that bulk CSF flow or absorption is *not necessary* for caudocranial drug dispersion to occur. Rather, experiments suggest that caudocranial transport of infused solutes can simply result from the asymmetry of the CSF spaces and graded CSF pulse amplitudes.

Recently, an interesting analytical solution for drug dispersion in the SAS was developed by Lawrence et al. (2019). Dispersion predicted inside an idealized annular geometry also found solute transport controlled by convection but not Taylor dispersion.

### Derivation of guidelines from *in vitro* experiments

Tracer dispersion after high-volume injection was very rapid, reaching the cervical region in less than 10 minutes, and it spread quickly throughout the spinal SAS. The apparent dispersion coefficient was robustly determined experimentally as a function of CSF amplitudes and frequencies. An empirical correlation (Eq. 8) between apparent diffusion coefficient and CSF pulsations, a function of CSF amplitude and oscillation, was established. This formula can be of clinical interest to predict tracer dispersion for IT drug administration.

We provide a simple guideline for estimating the volume of distribution of the drug during the injection phase as a function of catheter caliber and injection volume based on the experimental data and model in Eq. 5. Table 1 summarizes the expected size of the injection front (dispersion length after 10 min) from the lumbar injection site. It can also be used to get an idea about the advancement from the injection site toward the cranium, since the moving front of the tracer profile advances at least half of DD_10_.

**TABLE 1:** Guide for caudocranial motion, DD_10_, as a function of injection needle diameter (top row) and infusion volumetric flow rate (first column).

Caudocranial motion after 10 min from the injection site (cm)	d = 0.1 mm^b^	0.2 mm	0.5 mm	1.0 mm	1.5 mm	3.2 mm
0.25 mL/min^a^	20.49	20.34	19.92	19.21	18.51	16.10
0.50 mL/min	21.20	20.06	20.63	19.92	19.22	16.82
1.00 mL/min	22.62	22.48	22.05	21.35	20.64	18.24
2.00 mL/min	25.46	25.32	24.90	24.19	23.48	21.08
2.50 mL/min	26.88	26.74	26.32	25.61	24.90	22.50

^a^ Infusion volumetric flowrate.

^b^ Inner diameter.

For tracer dispersion in the oscillatory CSF flow (phase-2), Eq. 8 quantifies effective dispersion as a function of stroke volume and pulse frequency. Table 2 derived from these data enables the estimation of the effective dispersion coefficient based on physiological parameters. For drug molecules with different molecular diffusion coefficients (i.e., drugs with substantially different properties of our tracer), it may be used as a first approximation when no data are available or when its *Pe* number is in the same range as for trypan blue with a diffusion coefficient of D_0_ = 1.938 × 10^−6^ cm^2^/min.

**TABLE 2:** Table of clinical data, dispersion coefficient D.

D (cm^2^/min)		Stroke volume ml/bt^b^				
		0.25	0.5	0.7	1.0	1.5
Frequency bpm^a^	40	0.97	1.63	2.09	2.73	3.69
	60	1.29	2.17	2.78	3.63	4.91
	80	1.55	2.59	3.33	4.35	5.88
	100	1.67	2.79	3.59	4.68	6.33

a bpm—frequency of the CSF pulsation in beats per minute.

b ml/bt—stroke volume of the CSF flow in the cervical region.

### Comparison to Taylor–Aris dispersion theory

The analytical Taylor–Aris approach was shown to grossly overestimate the bio dispersion process. All versions of the TAD models showed that the spread of the tracer in human spine does not follow the trend proposed by Taylor dispersion. Effective dispersion by geometry-induced mixing seems to follow a different trend than that described by TAD. Moreover, TAD does not consider the effect of injection volume, catheter geometry, and placement as discussed during phase-1 of the infusion experiments. Also, TAD assumes a constant flow velocity throughout the channel. Finally, poor predictions by TAD theories for IT tracer dispersion and the very large Peclet number ranges seem to suggest that the molecular diffusivity (D_0_ in the denominator of Pe) may not be an ideal dimensionless scaling characteristic.

### Eccentricity and stagnation zones

Several authors have implicated the eccentricity of idealized cross-sectional areas of CSF-filled spaces in the spinal SAS as a key factor for accelerated drug dispersion (Loth et al., 2001; Sánchez et al., 2018; Chu et al., 2019; Moral-Pulido et al., 2023). The insensitivity to centric or eccentric alignment in our experiments does not seem to support the notion of eccentricity as a significant factor for the speed of IT dispersion. Moreover, we could not observe stagnation or recirculation zones in our experiments.

### Limitations

There are safety limits to high-volume injections adding the CSF amount during drug administration. We have experienced in rat that no more than 10% of the CSF volume can be safely injected over a period of a couple of minutes (Venugopal et al., 2017). Higher injection impulse may also contribute to the possibility of high shear rates that nerve roots experience near the catheter tip, which may again pose an additional risk that requires clinical investigation.

We did not investigate the dispersion of active drug molecules because we believe that tracers were ideal to quantify the physical transport phenomena with optical methods. We also did not consider biological functions such as the uptake in spinal or cerebral tissue because the focus was on determining dispersion coefficients as a function of physical flow phenomenon, amplitude and frequency of the CSF. Uptake studies would require a different setting. The *in vivo* study by Tangen et al. (2017b) could be a starting point, but parametric studies of CSF amplitude and frequency in humans are not practical, if not unethical. Therefore, the replica of the human CSF conditions seems a good choice for the parametric dispersion studies pursued here.

The biomechanical stress–strain relation of the epidural spaces is a function of several poorly understood factors. These include viscous resistance exerted by venous blood volume and fatty tissues, possible elastic deformation resistance of nerve roots bulging into peripheral distal spaces, and the biomechanical properties of the dura membrane including the stiffening effect of trabeculae and ligaments. Our system does neither consider CSF generation in the choroid plexus nor reabsorption (elimination) in the subarachnoid villi. These additional features could be incorporated in a more refined head model, which was not needed in our study focusing on spinal dynamics. The current model was able to induce cervical CSF displacements (stroke volume 0–1 mL/beat) within the physiological range but was not designed to faithfully reproduce the biomechanical compliance of the spinal compartment. Accordingly, we also did not attempt to measure the absolute pressure changes and gradients that occur during infusion. We plan to study the rise in the line pressure or the increase in pressure in the CSF of the spinal SAS as a function of injection parameters in future work.

Another related limitation pertains to the practice of fluid removal before high-volume injection (initial CSF tapping). There was no attempt made to interrogate the CNS model regarding the biomechanical response of the CSF spaces subjected to tapping.

## Conclusion

We conducted an extensive parametric study of tracer distribution in a subject-specific deformable bench model of the human CNS with anatomical and functional reproduction of CSF dynamics from the spine to the cranium. A systematic variation of parameters in numerous infusion experiments enabled the *in vitro* quantification of the spatiotemporal tracer dispersion patterns and their dependence on significant infusion parameters and pulsatile CSF conditions. This study reports a unique set of experimental data on the combined effect of infusion and natural oscillations in a deformable CNS replica that have not been reported previously. Our bench experiments suggest the feasibility of targeting large sections of the neuroaxis to the brain with the help of high-volume injection protocols. Infusion studies using human CNS models may serve as an inexpensive surrogate for testing and optimizing infusion protocols for the safe distribution of IT-administered solutes along the neuraxis to inform clinical trials in humans.

## Data Availability

The original contributions presented in the study are included in the article/Supplementary Material. Further inquiries can be directed to the corresponding author.
