# Supplementary material for: Determination of spinal tracer dispersion after intrathecal injection in a deformable CNS model
Source: Front Physiol. 2023 Sep 25;14:1244016. doi: 10.3389/fphys.2023.1244016 (PMC10561273; doi:10.3389/fphys.2023.1244016)
Supplement: Supplementary file 1 [file DataSheet1.pdf]

Determination of spinal tracer dispersion after intrathecal injection in a deformable CNS model

Frontier in Physiology – Computational Physiology & Medicine.

Ayankola O. Ayansiji[1,2], Daniel S. Gehrke[1], Bastien Baralle[3], Ariel Nozain[3],

Meenesh R. Singh[2] and Andreas A. Linninger[1,4*]

[1] Department of Bioengineering, University of Illinois Chicago, Chicago, Illinois, USA.

[2] Department of Chemical Engineering, University of Illinois Chicago, Chicago, Illinois, USA.

[3] UIC student intern from EPF, Ecole D'Ingénieur, Paris, France.

[4] Department of Neurosurgery, University of Illinois Chicago, Chicago, Illinois, USA.

Corresponding author:

Andreas A. Linninger, Department of Biomedical Engineering and Neurosurgery, University of Illinois at Chicago, Chicago, IL, USA.

Email: linninge@uic.edu

Phone (office): 312-4137743

ELECTRONIC SUPPLEMENTARY MATERIALS

Appendix A: Realization of cerebrospinal fluid pulsation due to vascular expansion

Pulsatile fluid motion was induced via an inflatable cerebrovascular compartment generating cerebrospinal fluid (CSF) flow patterns in the cervical, thoracic, and lumbar regions in a deformable spinal subarachnoid space (SAS) model that covered a physiological range of infusion volumetric flow rate (IVF) obtained previously in subject-specific magnetic resonance imaging CSF flow measurements.

Induction of CSF motion. Induction of pulsatile cerebrovascular expansion is controlled by a distensible balloon connected to a peristaltic piston pump, which puts in motion artificial CSF inside the spinal SAS. The peristaltic piston pump has two settings for the displacement volume moved with each stroke (0 to 1.0 mL) and allows for change in pulsation frequency (0 to 300 bpm). The pump has a set duration of stroke time. The vascular volumetric strain mainly serves to displace fluid from the cranial to the spinal CSF. Dilation of the vascular balloon can be adjusted using a peristaltic piston pump (Walchem E-Class Metering Pump) to control the range of expansion and contraction to generate desired levels of CSF stroke volumes (i.e., the additional volume of CSF introduced into the extramedullary space of the central nervous system model with each beat) of up to 1.0 ml/beat at oscillations from 0–180 beats per minute in the cervical region.

Injection system. Precise settings for bolus or continuous infusion volumes and timing were controlled with a syringe pump (Infusion Pump: NE-4000 Syringe Pump). Bolus volumes in the range of 1.0 mL and 2.0 mL of the trypan blue dye (0.1% and 0.4%-vol aq., Sigma-Aldrich) were selected. The exit velocity and kinetic energies for different needles and infusion volumetric flow rate is shown in table A1. During and after the infusion, CSF oscillations were maintained with the expanding vascular component (=piston pump) at 40, 72, and 120 beats per minute. These conditions were set to match the natural

dynamics of pulsatile CSF found with cine phase-contrast magnetic resonance imaging of the human cranium and spinal canal.

Table A1. The exit velocities (V_{Ext}) and kinetic energies (KE) for needle 1, needle 2, and needle 3 repeated for three different infusion volumetric flow rate (IVF).

Needle type	IVF ($mL.min^{-1}$)	V_{Ext} ($m.s^{-1}$)	KE ($kg.m^2.s^{-2}$)
Needle 1 (d= 0.2 mm)	0.5	0.2700	3.645×10^{-2}
	1.0	0.5300	1.405×10^{-1}
	2.0	1.0600	5.618×10^{-1}
Needle 2 (d= 1 mm)	0.5	0.0100	1.000×10^{-4}
	1.0	0.0200	2.000×10^{-4}
	2.0	0.0400	8.000×10^{-4}
Needle 3 (d= 3.2 mm)	0.5	0.0006	1.800×10^{-7}
	1.0	0.0011	6.000×10^{-7}
	2.0	0.0022	2.420×10^{-6}

Appendix B: Image data processing of the tracer infusion experiments

A high-speed video clip featuring the experiment preparation as well as the entire run covering 20 min of both injections. First, by determining the duration of the infusion (Phase-1) and the duration of the dispersion (Phase-2); one frame is a baseline reference (zero tracer present), and the rest are applicable for data extraction (tracer present). we obtained ten (10) snapshots from the recorded experimental video from the start of the Phase-2 at an interval of 1 minutes and stored them as image (in .bmp format) set in a file that is accessible to a code developed with MATLAB 2019b to quantify the dispersion in the Human Spine Replica (HSR). A high-speed video clip featuring the experiment preparation as well as the entire run covering 20 min of both injections. First, we load a custom program written in MATLAB 2019b to quantify biodispersion in the HSR from video recordings of the experiment. The video is imported as a '.mp4' format from the file directory. Select frames are chosen for analysis at predetermined time points covering the infusion (Phase-1) and the duration of the dispersion (Phase-2); one frame is a baseline reference (zero tracer present), and the rest are applicable for data extraction (tracer present). These unedited original frames are saved as a set. Next, a copy is made of the set and converted to a greyscale matrix. The difference of the greyscale images with tracer present to the baseline reference is saved as a third matrix and saved as a 1 or 0 to create a mask of the region of interest (ROI) in the original images where tracer is present. These frames (matrix pages) are now the masks respective of each frame time point. With the mask frame matrix, the software matches each greyscale difference frame to the original, unaltered image to select only the pixel red green blue (RGB) values of tracer in the original, unaltered frames using the mask ROI. A waterfall diagram of the final sequence of frames from the video recordings from the experiment is inferred from the software, which represents the mass flux of the tracer for each time point of phase-2 (Fig. B1). A calibration of known concentrations of trypan blue matched to the RGB values of images of the known concentrations

provides a sure fit of a concentration of trypan blue to a given RGB value within an image of tracer in the experiments. Extraction of the concentration value saved to a fourth matrix occurs only for the pixels of the original frames located at the mask ROI. Then, the software saves each frame of the pixel ROI determined from the mask matrix as an RGB to a fifth matrix. Each frame of the fifth matrix is computed for the mean RGB value for each row of the matrix. The semi-automatic program in MATLAB calculates the likely concentration with the polynomial fit. Normalization is performed to ensure the mass flux represented by the integral area of the curve remains constant for each curve, and the results are plotted and saved to a '.mat' file for further analysis.

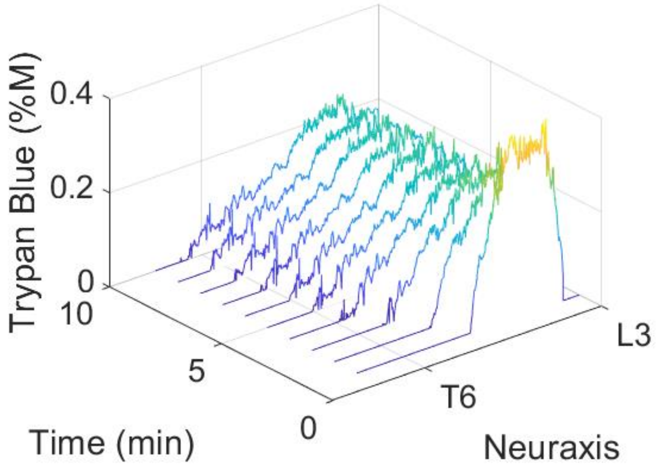

Fig. B1. The waterfall represents the mass flux of the tracer for each time point of phase-2.

Appendix C: Dispersion Front Tracking and Analysis.

The RGB values in the range of [0, 255] were acquired from bio dispersion recorded video at different times. The acquisition of the RGB data followed two modes: (i) We scanned RGB data at the center of the snapshots at different time points as shown in the figure C1. (ii) We also used vertical bands of up to 5 pixels that spanned the clear fluid space in radial dimension. Both methods gave essentially the same profiles as shown in Fig. C1 and Fig. C2. The RGB values associated with each position along the neuraxis were then converted to grayscale data using Eqs. (C1 and C2). Note that the method of moments does not require absolute concentrations but is robust against imaging artifacts it only tracks the slope of the temporal evolution of the mean and variance of the entire, spatially distributed intensity curves.

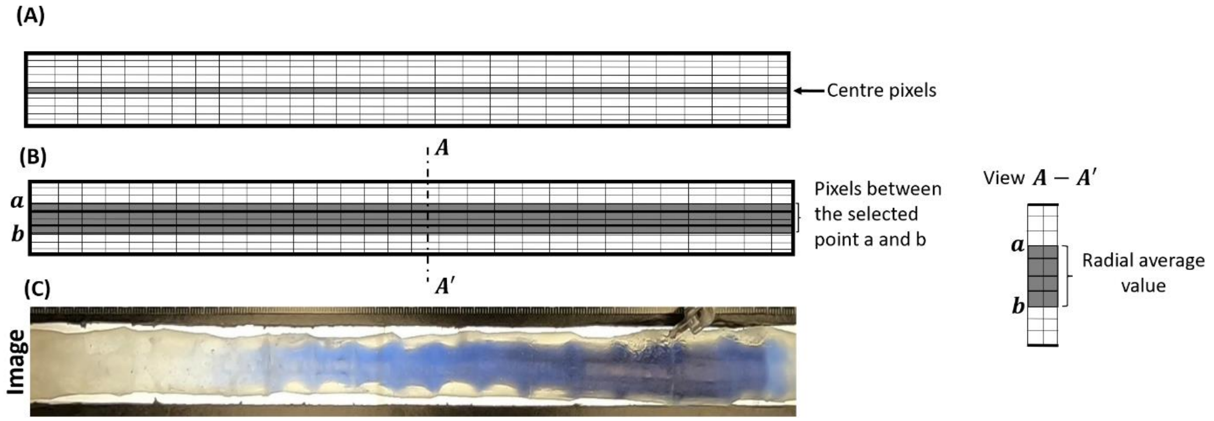

Fig. C1. (A) When the RGB data in the center pixels are analyzed. (B) When RGB data in the pixels between the selected points a and b are analyzed and averaged in radial direction (C) Sample of the raw image data obtained on which pixels and RGB data are obtained.

Another approach, as shown in Fig. C1. B, was also used which is the averaging over a selected pixels in the vertical columns (radial direction). The same results were observed in both cases. The RGB data obtained using the two approaches are plotted in Fig. R2 below. The curve overlap which shows that there is no noticeable difference in both methods.

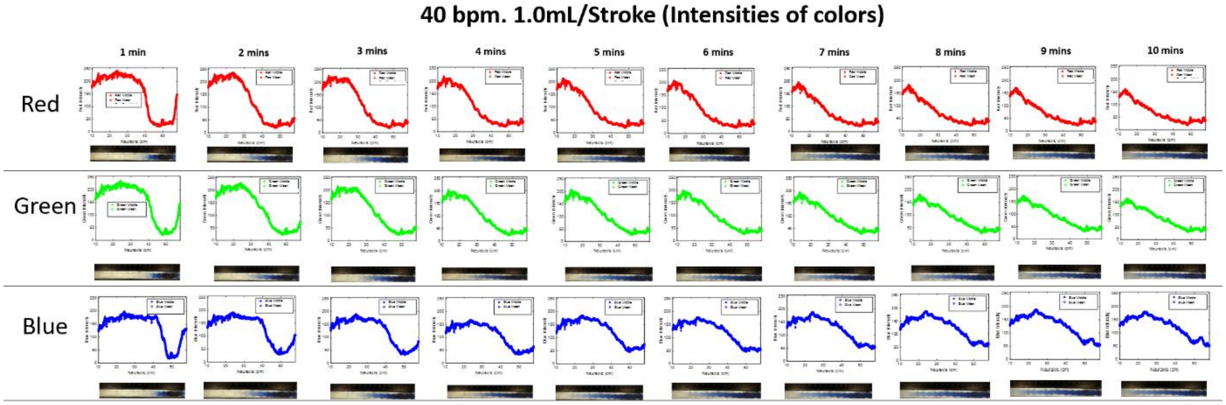

Fig. C2. Figure showing the plot of the RGB intensities using both methods (Fig. C1 A and Fig. C1 B) described above

To track and analyze the dispersion front of the experiment, two methods (grayscale formula and grayscale binary) were used. Below are the steps taken for the two methods:

Grayscale formula

- The RGB extraction from the frame. The RGB ranges from 0 to 255.
- With the use of the gray scale formular, intensities were obtained.

$$GrayScale\ (y)\ =\ S_1\ R + S_2\ G + S_3\ B \tag{C1}$$

Where: $S_1 = 0.2989$, $S_2 = 0.5870$, $S_3 = 0.1140$,

$R = Red\ channel$, $G = Green\ channel$, and $B = Blue\ channel$.

- White offset was done, using the inversion formula, to get the concentration profile.
- The concentration profiles were plotted for each frame and scaled to have the maximum concentration as 0.4 (%M).
- Area under the curves is constant to ensure that mass is conserved.
- Second moments were computed, and the half of the gradient of the second moment plot gives the dispersion coefficient

Conversion formula from intensity to tracer concentration needed in Method of Moments (MoM).

Note that knowledge of the absolute concentration is not needed in the MoM, although this can be done by calibration [A1].

$$y_{inv} = y_{max} - y_i + y_{min} \tag{C2}$$

Where y is the intensity curve

y_{max} = maximum value of the intensity curve

y_{min} = minimum value of the intensity curve

y_i = the intensity level y at each position i

y_{inv} = the inferred intensity (concentration) at a point along the neuraxis

Fig. C3 shows the evolution of the tracer using the grayscale formula for experiment 40bpm and 72bpm at 1.0mL/Stroke. Figure C5 shows the plots of the first moment for all the experimental data. To have clean image data, a white florescence light was used with a white canvas for background control. All experiments were run three times to ensure reproducibility. The low variation in the results obtained for each three experiments shows that the study is reproducible.

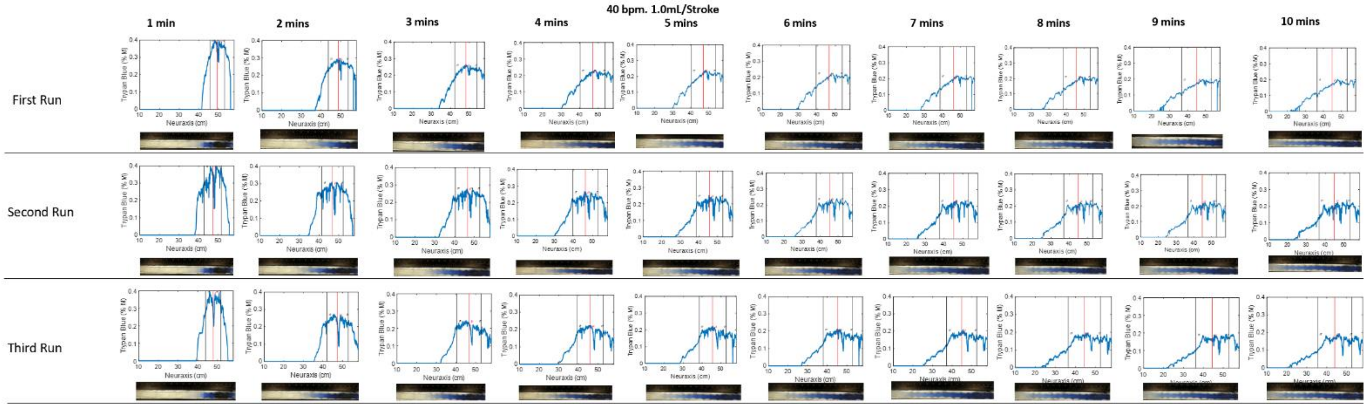

C_Inferred= $S_1\ R + S_2\ \mathrm{G} + S_3\ B$ $S_1 = 0.2989$ $S_2 = 0.5870$ $S_3 = 0.1140$ **Using the Gray formular**

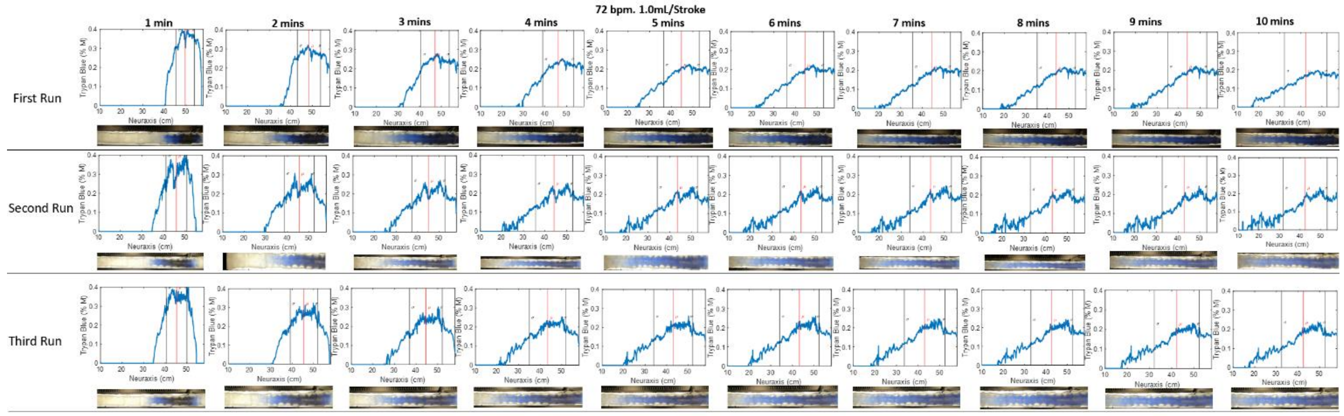

C_Inferred= $S_1\ R + S_2\ \mathrm{G} + S_3\ B$ $S_1 = 0.2989$ $S_2 = 0.5870$ $S_3 = 0.1140$ **Using the Gray formular**

Fig. C3. The evolution of the tracer in the domain using the grayscale formula for experiment 40bpm and 72bpm both at 1.0mL/Stroke. It must be noted that the white offset has been done to ensure that the concentration profile pattern is correct. Also, the area under the curves is constant to ensure accuracy of the analysis.

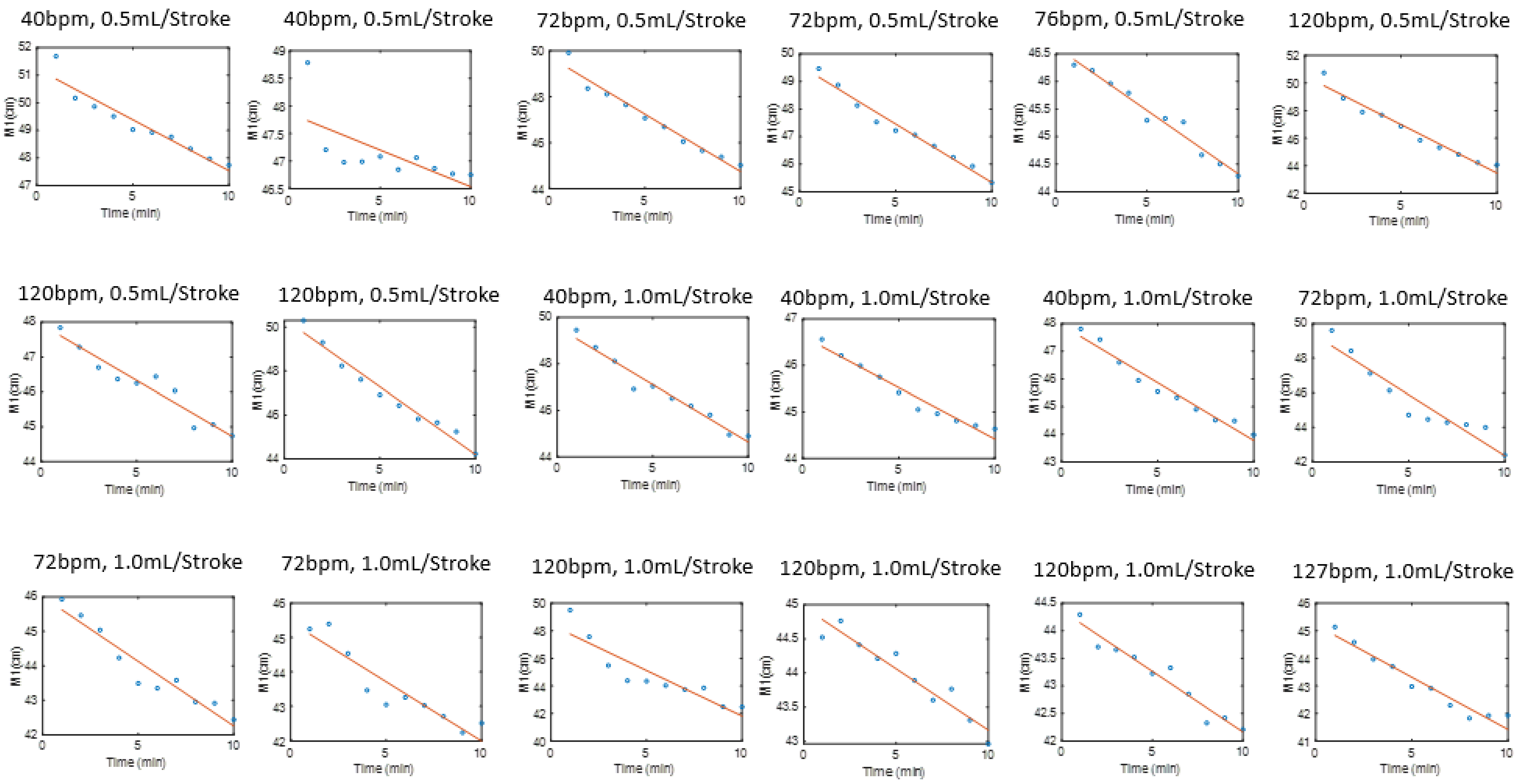

Fig. C4. First moment plots for all the experimental data. The first moment, M1, indicates that the tracer moves in cranial direction from the injection position (50 cm) towards the thoracic region position (44 cm).

Binary (0-1) Image analysis. Alternative method not requiring quantitative intensity information at all. This method could be used on qualitative (binary) immunobiological essays.

- The RGB extraction from the frame
- With the use of the gray scale formular as shown in Eq. (C1), intensities were obtained.
- Binary method was applied to assign the concentration of 0.4 (%M) form region where there is tracer (i.e., where the value of the gray data is 1). 0 (%M) was assigned to the region where gray data is 0.
- The concentration profiles were plotted for each frame
- Area under the curves is constant to ensure that mass is conserved.
- Second moments were computed, and the half of the gradient of the second moment plot gives the dispersion coefficient.

Fig. C5 shows the evolution of the tracer using the binary method for experiment 40bpm and 72bpm at 1.0mL/Stroke.

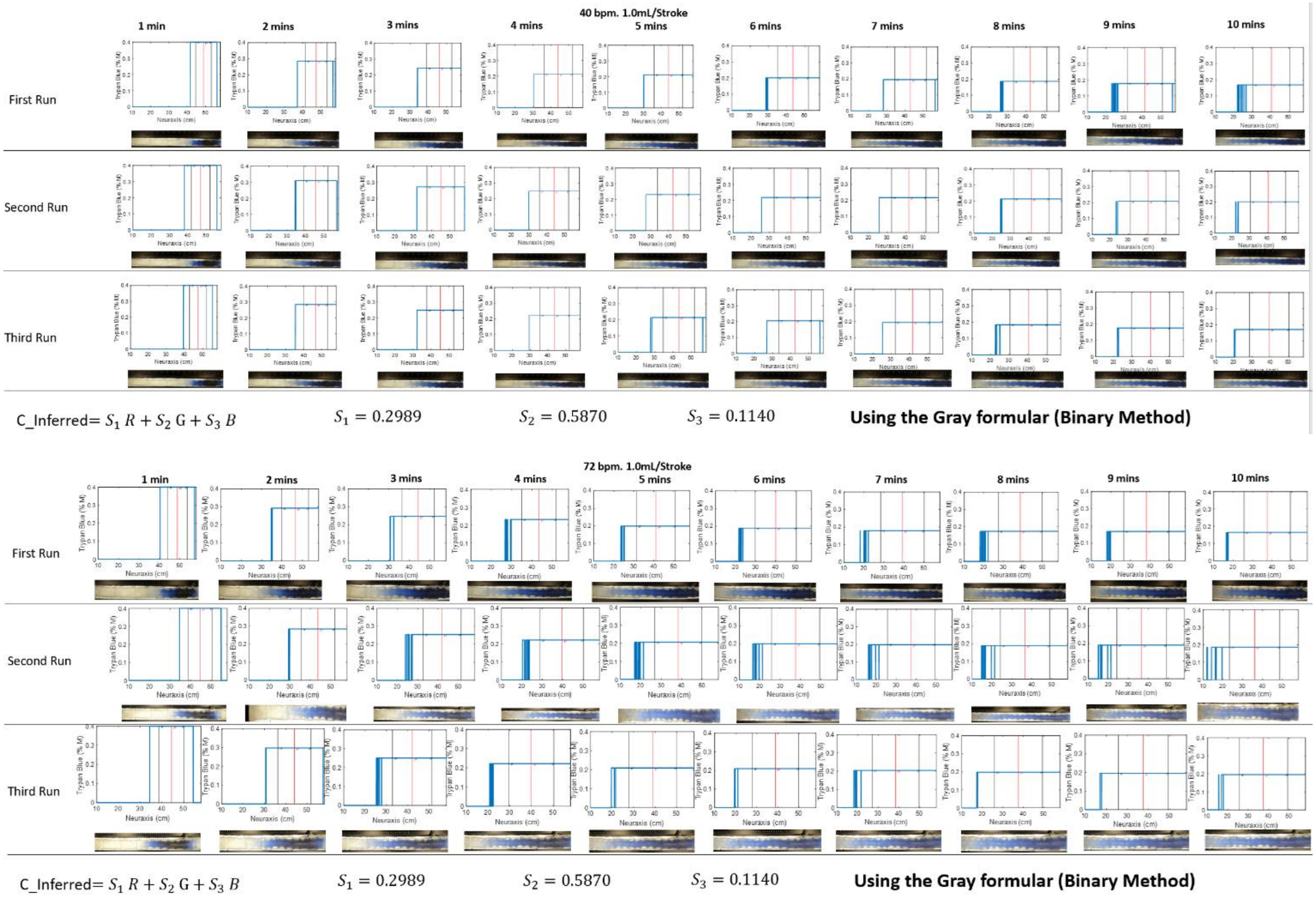

Fig.C5. The evolution of the tracer in the domain using the grayscale formula (Binary) for experiment 40bpm and 72bpm both at 1.0mL/Stroke. The area under the curves is constant to ensure accuracy of the analysis.

Appendix D: Computation of dispersion coefficient

The dispersion coefficients were obtained from the plot of second moment data with time. The half of gradient of the curve obtained through linear regression gives the dispersion coefficient. Consider the second moment plot in fig. D1.

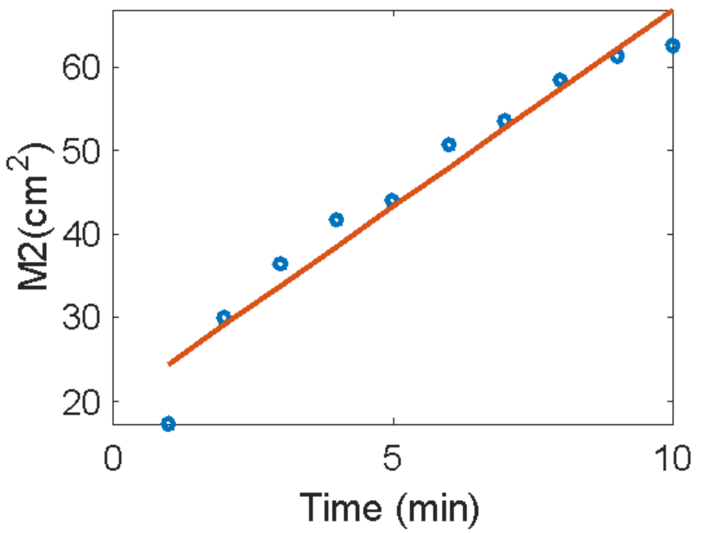

Fig. D1. The second moment plot for the system with frequency of 72bpm and stroke volume of 0.5mL/stroke

Using linear regression, M2 = a + b t. Where M2 is the second moment in cm^2, t is the time in minutes, and b is the gradient. The half of gradient of the curve is 2.36 which gives the dispersion coefficient in cm^2/min.

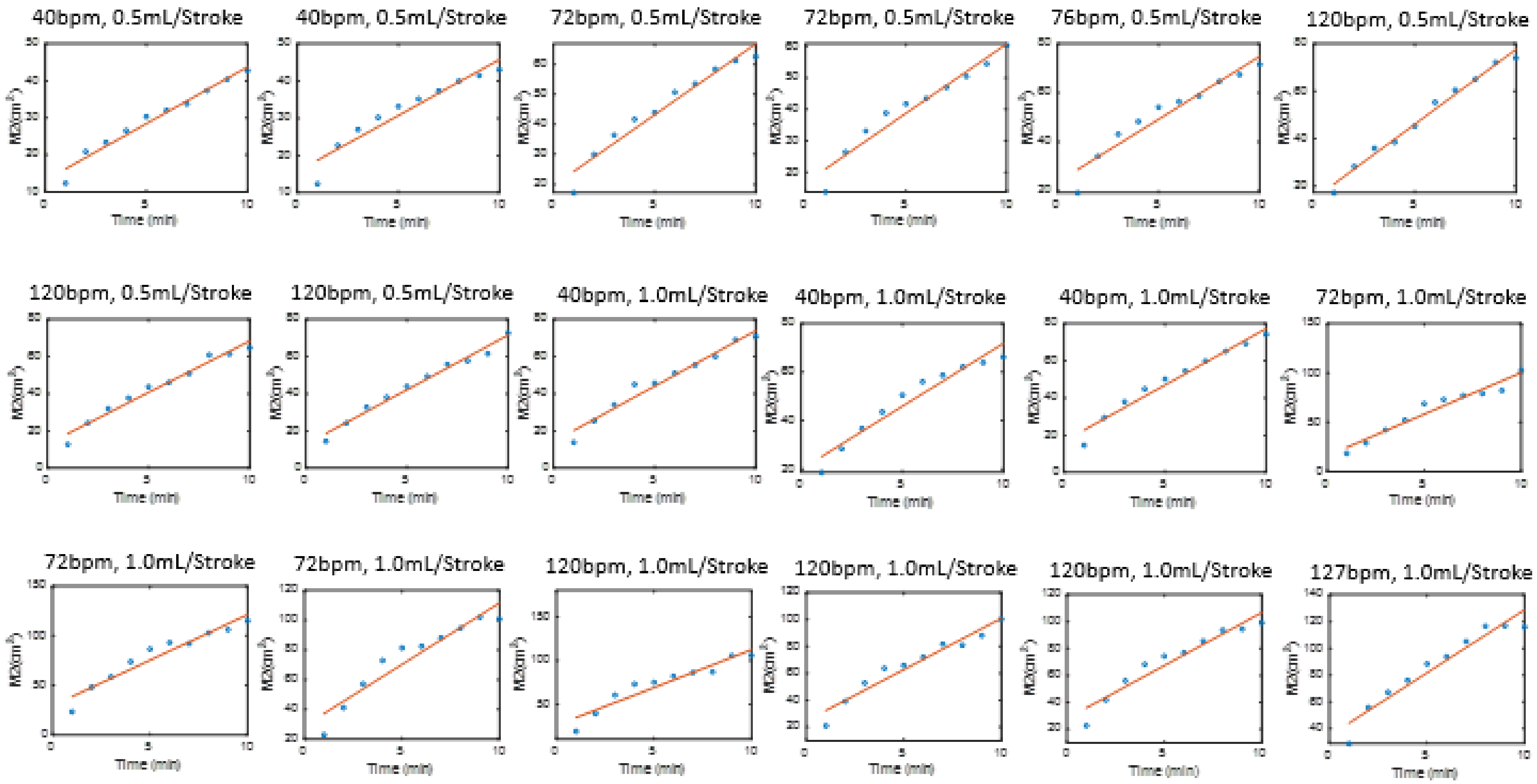

Fig. D2. Second moment plots for all the experimental data which shows the gradients of the second moment with which the dispersion coefficients are obtained.

Appendix E: Detail computation of the dispersion coefficient using Taylor-Aris Dispersion (TAD) analysis.

Consider the Fig E1 which shows the schematic diagram for the bio-dispersion process in a deformable spine of length, l, height, h, and width, w. The schematic shows the three regions of the spine, I.e., cervical, thoracic, and lumbar regions. There is pulsating flow in the domain with stroke-volume as shown in the figure. The infusion point shows the point where the tracer was introduced to the pulsating flow the system. No flow out of the system. In the Fig E1, the stroke-volume model shows that there is change in the volume of the fluid in the system as fluid moves in and out of the system with V being the initial volume of fluid in the system and dv being the change in the volume of fluid in the system.

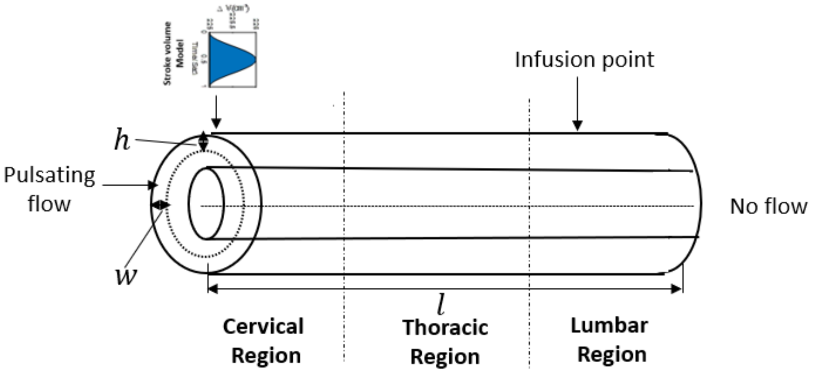

Fig E1. The schematic diagram for the bio dispersion process

Overview of experimental dimensions

Using the experimental data of frequency, stroke volume, height, and width of the domain**,** we computed for each experiment dimensionless characteristic numbers, Peclet number (Pe), and Womersley number (α). We determined the dispersion coefficient experimentally with the method of moment and listed each dispersion coefficient in the context of the dimensionless experimental settings. We plotted the experimental dispersion coefficient as a function of frequency and stroke volume. We also plotted the experimental dispersion coefficient as a function of Peclet number and Womersley

number. The description of how to compute the Peclet number is given in Eq. (E1). The major term in Eq. (E1) is the root-mean-square velocity ($U_{r.m.s}$) of the solute. Considering that the domain of study is deformable and there is no flow out of the domain, the velocity of the solute is attenuated from the cervical region to the lumbar region. In our study, the mean velocity used for computation is that at the cervical region which is the maximum velocity in the domain. Table E1 shows the parameter used for the computation of the dimensionless quantities.

Table E1: The table of the parameter used for the computation.

Parameter	Values	Units
Height (h)	0.25	cm
Width (w)	0.15	cm
Length (l)	65	cm
Fluid Content (V)	225	mL
Hydraulic Cross-Section	0.13	cm^2
Diffusivity of Trypan Blue (D_0)	1.938×10^{-6}	cm^2/min

The Peclet number is computed as Eq. (E1)

$$Peclet\ number = \frac{U_{r.m.s}\left(\frac{cm}{min}\right) * Height\ (cm)}{Diffusivity\ of\ Trypan\ Blue\ \left(\frac{cm^2}{min}\right)} \tag{E1}$$

Where $U_{r.m.s}$ is the root-mean-square velocity. The values for the Peclet number are shown in table E2 and table E3 for different velocities. The $U_{r.m.s}$ of the CSF was obtained using the different stroke volumes at different frequencies. The stroke volume model used is shown in Eq. (E2). For the stroke volume of v_c mL/stroke, the stroke volume conversion to Total volume is shown in Eq. (E2)

$$V(t) = V_0 + \left(\frac{v_c}{2}\right) - \left(\frac{v_c}{2}\right) \cos(\omega\ t) \tag{E2}$$

$$\omega = \frac{2\pi}{60} * N_{bpm}$$

Where V is the total volume change in cm^3, V_0 is the initial volume of CSF in cm^3 in the system, ω is the angular frequency, $N_{b.p.m}$ is the frequency in beat per minute, and t is the time in seconds.

The volume change was modeled as a function of time using cosine function which later converted to the instantaneous volume change through the differentiation of the volume change. Using cross-section area of the spine at the cervical region, the instantaneous volume change was converted into the velocity of the CSF at the cervical region. Finally, the root-mean-square of the obtained velocity was obtained which was used in the computation of the Peclet number.

It must be noted that the root-mean-square velocity varies along the length of the spine with its value as zero at the sacral which is as result of the deformation of the spine. In our analysis of the TAD, we used the highest $U_{r.m.s}$ in the spine which is obtained at the cervical region. This is to estimate the highest dispersion that could be obtained in the system at different stroke volumes and different frequencies.

The Womersley number [A2], $\alpha = \frac{D_H}{2}\sqrt{\frac{\omega}{\upsilon}} = h\sqrt{\frac{\omega}{\upsilon}}$. $D_H = 0.5cm$, is the hydraulic diameter, $\omega = \frac{2\pi f}{T}$, f is the frequency, $T = 1$ sec, is the period of oscillation, and $\upsilon = 7x10^{-7}m^2/sec$, is the kinematic viscosity.

Table E2 and table E3 show the dispersion coefficients data for stroke volume of 0.5mL/Stroke and 1.0mL/Stroke respectively. The tables also show the data for dispersion coefficient using Taylor approach as shown in Eq. (9) in the main text.

From table E2 and table E3, some of the experiments were repeated for some frequencies. Even though variance exists, what we can see is that the trend is clear.

Table E2 shows the frequency, root-mean-square velocity, Pe, dispersion coefficient, and **α** for 0.5mL/Stroke.

**Frequency (bpm)**	**U_{rms} (cm/min)**	**Pe (10^6)**	**Dispersion Coeff (cm^2/min) (Exp)**	**Dispersion Coeff (cm^2/min) 10^4 (Taylor)**	**α**
40	6.6	0.8514	1.53<br>1.50	0.2838	6.12
72	12.0	1.5480	2.36<br>2.20	0.8733	8.20
76	12.6	1.6254	2.57	0.9572	8.43
120	19.8	2.5542	3.15<br>2.77<br>2.94	2.2388	10.59

Table E3 shows the frequency, root-mean-square velocity, Pe, dispersion coefficient, and **α** for 1.0 mL/Stroke.

**Frequency (bpm)**	**U_{rms} (cm/min)**	**Pe (10^7)**	**Dispersion Coeff (cm^2/min) (Exp)**	**Dispersion Coeff (cm^2/min) 10^4 (Taylor)**	**α**
40	13.2	1.7028	2.98<br>2.57<br>3.03	1.0446	6.12
72	24.0	3.0960	4.22<br>4.59<br>4.13	3.2143	8.20
120	39.6	5.1084	4.30<br>3.81<br>3.94	8.2405	10.59
127	42.0	5.4180	4.70	9.2045	10.90

An empirical correlation Eq. (E3) between apparent diffusion coefficient and CSF pulsations, a function of CSF amplitude and oscillation was established as a function of the root-mean-square velocity of the CSF, measured in the cervical region, as well as the frequency of the CSF pulsations. Where $\lambda(f) = a_0 + a_1 f + a_2 f^2$. $a_0 = -1.0386,\ a_1 = 0.0055,\ and\ a_2 = -5.8858 \times 10^{-5}$ are constant terms, and κ = 0.7419. U_{rms} is the root-mean-square velocity of the CSF, f is the frequency and D_{exp} is the experimental dispersion coefficient for the dimensional model.

$$\log(D_{exp}) = \lambda(f) + \kappa \log(U_{rms}) \quad \text{(E3)}$$

Dimensionless form of Eq. (E3) was also developed where the dispersion coefficient is a function of Pe and A. This was done by obtaining the Pe and the α at each state as used in Eq. (E3) then fitted using linear model as shown in Eq. (E4).

$$\log(\Delta\mathfrak{D}) = \lambda(\alpha) + \mathrm{K} \log(Pe) \quad \text{(E4)}$$

Where α is the Womersley number, $\alpha = \frac{D_H}{2}\sqrt{\frac{\omega}{\upsilon}}$, D_H is the hydraulic diameter, $\omega = \frac{2\pi f}{T}$, f is the frequency, T is the period of oscillation, and υ is the kinematic viscosity, $\lambda(\alpha) = \varphi_0 + \varphi_1 \alpha + \varphi_2 \alpha^2$, $\varphi_0 = 2.4786,\ \varphi_1 = 0.3130,\ \varphi_2 = -0.0229$, and $\mathrm{K} = 0.7400$ are constant terms, $\Delta\mathfrak{D} = \frac{D_{exp}-D_0}{D_0}$, Pe is the Peclet number, and D_0 is the molecular diffusion coefficient.

Table E4 shows the comparison of the experimental obtained dispersion coefficient to the coefficients obtained by Taylor approach and the fitting models (dimensional, and dimensionless) for phase 2.

**Stroke Volume**	**Freq (bpm)**	**Exp**	**Dimensional**		**Dimensionless**	
		D **(cm²/min)**	$\boldsymbol{D_{exp*}}$ **(cm²/min)**	$\boldsymbol{\frac{\lvert D - D_{exp*}\rvert}{D}}$ **(%)**	$\boldsymbol{D_{exp*}}$ **(cm²/min)**	$\boldsymbol{\frac{\lvert D - D_{exp*}\rvert}{D}}$ **(%)**
	40	1.52±0.015	1.63	7.24	1.63	7.24
**0.5 mL / Stroke**	72	2.28±0.08	2.45	7.46	2.44	7.02
	76	2.57	2.51	2.33	2.50	2.72
	120	2.95±0.18	2.69	8.81	2.70	8.47
**1.0 mL / Stroke**	40	2.86±0.29	2.72	4.90	2.74	4.20
	72	4.31±0.18	4.10	4.87	4.09	5.10
	120	4.02±0.21	4.51	12.19	4.53	12.69
	127	4.70	4.42	5.96	4.38	6.81
			**Avg. %**	**6.72**	**Avg. %**	**6.78**

Where D_{exp*} is the recomputed dispersion coefficient using the dimensional and dimensionless models.

Statistical Analysis

The following are the statistical analysis (Sum of squared residuals (SSR), the coefficient of determination (R^2) and the variance (V)) of the models as shown in table E5.

Table E5: The statistical analysis of Eq. (E3) and Eq. (E4)

Model:	$\log(D_{exp}) = \lambda(f) + \kappa \log(U_{rms})$	$\log(\Delta\mathfrak{D}) = \lambda(\alpha) + \mathrm{K} \log(Pe)$
SSR	2.0059	2.0059
R^2	0.9273	0.9278
V	0.1114	0.1114

Appendix F: Stockman's dispersion coefficients compared to experimental data.

Fig. F1 shows our experimental data (also listed in Fig. 6b) in comparison to Stockman's theoretical predictions. The Stockman models used maximum average speed in a period of oscillation of 220.2cm/min (in lattice unit of $1x10^{-2}$ Lu/ts), and the molecular diffusion of the tracer used is D_0=0.0024cm^2/min [A3]. The dispersion coefficient was determined by Stockman with values of D=0.6356 - 1.7735cm^2/min which is much smaller than those of our experiment.

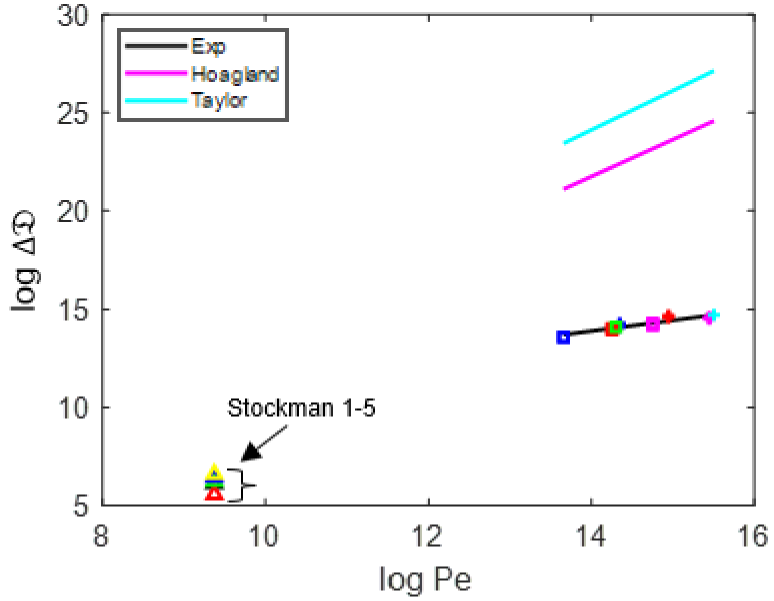

Fig. F1. Figure showing the comparison of the dispersion change against Peclet number between the experiment data, and the prediction by Taylor approaches. The blue, red, green, magenta, and cyan colored data points are for the frequencies of 40, 72, 76, 120, 127 bpm respectively. The data represented as square are for 0.5mL/Stroke and that represented with + are for 1.0mL/Stroke. Where Stockman 1 – Stockman Spinal Model D, Stockman 2 – Stockman Model B-Normal, Stockman 3 – Stockman Model B-Dense, Stockman 4 – Stockman Model D-Normal, Stockman 5 – Stockman Model D-Dense.

Appendix References

[A1] O. Ivanchenko, N. Sindhwani, and A. Linninger, “Experimental Techniques for Studying Poroelasticity in Brain Phantom Gels Under High Flow Microinfusion,” *J. Biomech. Eng.*, vol. 132, no. 5, Mar. 2010, doi: 10.1115/1.4001164.

[A2] J. R. Womersley, “Method for the calculation of velocity, rate of flow and viscous drag in arteries when the pressure gradient is known,” *J. Physiol.*, vol. 127, no. 3, pp. 553–563, Mar. 1955.

[A3] Stockman HW. Effect of anatomical fine structure on the dispersion of solutes in the spinal subarachnoid space. J Biomech Eng. 2007;129(5):666–75.
